# Paralog transcriptional differentiation in the *D. melanogaster-*specific gene family *Sdic* across populations and spermatogenesis stages

**DOI:** 10.1038/s42003-023-05427-4

**Published:** 2023-10-20

**Authors:** Bryan D. Clifton, Imtiyaz Hariyani, Ashlyn Kimura, Fangning Luo, Alvin Nguyen, José M. Ranz

**Affiliations:** Department of Ecology and Evolutionary Biology, University of California Irvine, Irvine, CA 92697 USA

**Keywords:** Evolutionary genetics, Gene expression

## Abstract

How recently originated gene copies become stable genomic components remains uncertain as high sequence similarity of young duplicates precludes their functional characterization. The tandem multigene family *Sdic* is specific to *Drosophila melanogaster* and has been annotated across multiple reference-quality genome assemblies. Here we show the existence of a positive correlation between *Sdic* copy number and total *e*xpression, plus vast intrastrain differences in mRNA abundance among paralogs, using RNA-sequencing from testis of four strains with variable paralog composition. Single cell and nucleus RNA-sequencing data expose paralog expression differentiation in meiotic cell types within testis from third instar larva and adults. Additional RNA-sequencing across synthetic strains only differing in their *Y* chromosomes reveal a tissue-dependent *trans-*regulatory effect on *Sdic*: upregulation in testis and downregulation in male accessory gland. By leveraging paralog-specific expression information from tissue- and cell-specific data, our results elucidate the intraspecific functional diversification of a recently expanded tandem gene family.

## Abstract (web summary)

RNA-seq in fly lines with different *Sdic* paralog composition reveals paralog expression differentiation within and between lines, and across spermatogenesis stages, showing early functional diversification of recently expanded tandem gene families.

## Introduction

Despite the relevance of gene duplication in the evolution of genomes, adaptation, and phenotypic diversification^1–5^, the evolutionary steps underlying their integration and long-term retention in the species gene repertoire remain controversial^6,7^. A proposed mechanism is that gene duplicates might produce adaptive increases in gene product^8^. Another is that gene duplicates may act as material for evolutionary tinkering that eventually generates functionally differentiated paralogs^9,10^. This second scenario applies preferentially to RNA-based and defective duplicates as opposed to full DNA-based duplicates, as the latter have a lower probability to evolve new functional attributes due to their initially identical *cis*-regulatory sequences^11–13^. Overall, the underlying causes that explain paralog retention and functional divergence of gene duplicates as they age remain poorly understood^7,8,14–16^.

Recently generated duplicates, i.e., those still segregating or recently fixed, can substantially contribute to fill this gap in knowledge^17–20^. The reason is that, as the number of mutations between paralogs increases during evolutionary time, so does the number of mutations in the rest of the genome, including those affecting genes that are functionally related to the former. Consequently, the functional and phenotypic repercussions of mutations affecting gene duplicates become intertwined with those additional mutations, complicating the analysis of those directly affecting the paralogs. Unfortunately, although massively parallel short-read sequencing and microarray technologies have been highly informative about population trends underlying copy number (CN) variation, and illustrated the complex relationship between CN changes and alterations of expression levels^18,19,21,22^, they have fallen short in providing accurate information about paralog-specific sequence and expression differences, especially for young tandem gene expansions. Reasons for this refractoriness include that young tandem duplicates exhibit typically high sequence identity and that these structurally complex genomic regions are often improperly assembled even in reference genome assemblies^23,24^. Consequently, to faithfully decipher the early stages of paralog functional divergence, it is advisable to analyze recent tandem expansions whose annotation has been accurately resolved across individuals, which requires long-read sequencing-based assemblies^20,25^.

The tandem gene family *Sdic* (for *Sperm-specific dynein intermediate chain*) is unique to a single animal species, *D. melanogaster*^26,27^, highly expressed in testis, and known for its impact on male fertility through sperm competition^26,28,29^. The original *Sdic* copy originated from a segmental duplication involving two adjacent genes, *short wing* (*sw*) and *Annexin B10* (*AnxB10*), ultimately creating a defective but functional copy of *sw*^26^. Based on commonalities at the amino acid level with the sw protein, and sw’s role as part of a multiprotein motor complex^30^*, Sdic* is thought to code for a similar protein complex subunit. The *Sdic* region has been accurately reconstructed and annotated at the level of individual paralogs across a set of strains from different geographic origins^25^. The region exhibits CN variation (mostly 3–6 copies), and harbors only one paralog consistently present across strains^25^. From the start of the promoter to the STOP codon, the nucleotide sequence identity among paralogs is ~99%, with none showing evidence of pseudogenization at the sequence level^25,27^. Notably, and coincidental with previous genome-wide surveys^19,22,31^, no correlation between *Sdic* CN and expression level of the entire *Sdic* gene family was found, pointing toward regulatory variation—probably in *trans*—as the most relevant factor shaping its naturally occurring expression variation^25^. Nevertheless, expression profiling was done with whole bodies^25^, arguably masking tissue-specific expression differences^32^, and rendering unclear whether a similar buffering of transcript expression exists at the tissue level. Equally important, no expression data was obtained from individual paralogs, so the contribution of each paralog to the total *Sdic* expression remains elusive. Additionally, the expression of individual *Sdic* paralogs throughout spermatogenesis during the male life cycle is unknown. In sum, the *Sdic* multigene family offers a powerful system to gain key insight into the early evolutionary stages of the expression diversification of recently evolved tandem paralogs.

Here, we analyze the patterns of differentiation of several expression attributes among *Sdic* paralogs. First, using testis RNA-seq data from a geographically diverse set of strains for which the *Sdic* region has been properly assembled, we assessed whether total *Sdic* transcript abundance correlates with *Sdic* CN. In addition, we quantify differential expression of individual paralogs within these strains, examining how paralogs have evolved differences in mRNA abundance and in contribution to total *Sdic* expression. Next, by reanalyzing single-cell (sc) and single-nucleus (sn) RNA-seq data from third-instar larva and adult testis^33,34^, we tracked paralog-specific spatial expression across different stages of spermatogenesis, investigating emerging differences. Lastly, and due to the genome-wide *trans*-regulatory effect of the *Y* chromosome in both male reproductive and somatic tissues^35–37^, we generated a set of *Y* chromosome substitution lines with an otherwise identical genomic background in which we evaluated the impact of this chromosome on *Sdic* mRNA abundance in testis and male accessory gland, a somatic tissue part of the male reproductive system. This work showcases the importance of having accurately annotated paralogs within structurally complex genomic regions, as well as the use of tissue- and cell-specific expression data, to understand more precisely the initial stages of functional diversification among recently evolved tandem gene expansions.

## Results and discussion

### Total *Sdic* mRNA abundance positively correlates with *Sdic* copy number in testis

Evidence for the presumed enhancing effect of gene duplication on the amount of gene product is mixed. Some studies indicated that no such, or a very limited, effect exists^19,38–41^, whereas others found a significant increase^17,18,22,42,43^. A possible explanation for a limited effect on mRNA abundance is the relatively old age of the paralogs studied or the inclusion of incomplete duplicates with sometimes different promoters^42^. In fact, engineered duplications^16,25,44,45^ or mutation-accumulation experiments^46^ have shown that the immediate effect of gene duplications is elevated expression. Subsequently, different factors such as efficiency of selection—which depends on the species effective population size—, ancestral expression level, genomic background where the duplication arose, and whether the encoded product is part of multiprotein complexes, will impact paralog expression levels^42,45,46^. Importantly as well, most of the above studies focused on two tandem duplicates or were underpowered for different reasons, including the inability to distinguish between the expression levels of different paralogs, or the use of whole-body samples instead of tissues for expression profiling.

**Fig. 1:**
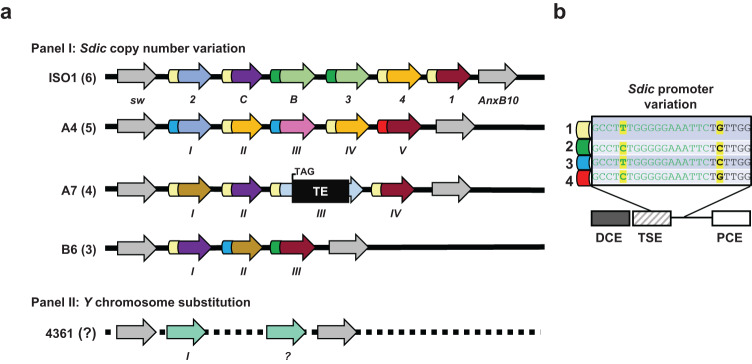
Structural and sequence diversity of the *Sdic* region. **a** Organization of the *Sdic* region on the *X* chromosome of *D. melanogaster* in two panels of strains. Strain name and the number of *Sdic* copies in each region are indicated. The region is shown as it is arranged from the centromere (left) to telomere (right). Each *Sdic* repeat consists of a transcriptional unit, a defective pseudogene of the flanking gene *AnxB10*, and a partial copy of the TE Rt1c (Fig. 1 in ref. ^27^). Only the transcriptional unit of *Sdic* is represented here. For the strain ISO1, the *Sdic* paralogs (arrows) are labeled as in FlyBase^80^, although adopting an improved annotation^27^. For other strains, the paralogs are identified using roman numerals following their order from *sw* to *AnxB10,* i.e. from centromere to telomere. In addition, the paralogs are color-coded based on the version of Sdic protein encoded as previously established^25^, thus reflecting different *Sdic* paratypes. The only paralog present in all strains is shown in dark-red color, being referred generally to as *Sdic1-*like outside ISO1, where it is called *Sdic1*. Promoters are also color-coded according to (**b**). Except for the paralog *III* in A7, any *Sdic* paralog is approximately ~5000-nt long (measured from the start of the promoter to the end of the 3’UTR). *SdicIII* in A7 contains a transposable element (TE) insertion that induces a premature stop codon. **b** *Sdic* promoter sequence variation. Two nucleotide sites are variable across the 18 promoters aligned, resulting in four types (1–4). Different sequence elements in the *Sdic* promoter are labeled as previously established^26^. DCE distal core element, TSE testis-specific core element, PCE proximal core element.

In the case of *Sdic*, previous qRT–PCR surveys of expression variation across six isogenic strains differing in geographical origin and CN showed that CN differences were not positively correlated with total *Sdic* expression, i.e., the expression level when all *Sdic* copies are surveyed jointly^25^. The assay performed used whole bodies and relied on primers designed within a fraction of the coding sequence with no nucleotide variation across paralogs both within and between strains. To test whether this apparent buffering effect in whole bodies is reproducible at the tissue level, we performed RNA-sequencing in testis, the tissue in which *Sdic* shows the highest expression level as confirmed by the reanalysis of 122 RNA-seq datasets from different sources, including FlyAtlas2 (Supplementary Figs. 1 and 2 and Supplementary Data 1). For that, we used 5 d old males from four isogenic strains (Panel I in Supplementary Tables 1 and 2), all of them possessing a reference-quality genome assembly^47,48^ in which the paralog composition and sequence of the *Sdic* region have been comprehensively resolved^25^ (Fig. 1a). *Sdic* paralogs differ at the nucleotide level both within and between strains, with most present as unique copies within strains^25,27^. Nevertheless, the first exon of *Sdic*, which evolved de novo from *sw* intergenic region, is identical in sequence across the paralogs and strains characterized so far and, crucially, it is absent from the coding region of the parental gene *sw*, thus detecting the expression of all *Sdic* paralogs. We leveraged this fact and implemented a conservative computational pipeline that screens sequencing reads, requiring the detection of a complete sequence motif unique to a given nucleotide sequence of interest, a section of the first exon of *Sdic* in this case^25,27^ (“Methods”; Supplementary Figs. 3 and 4 and Supplementary Table 3).

**Fig. 2:**
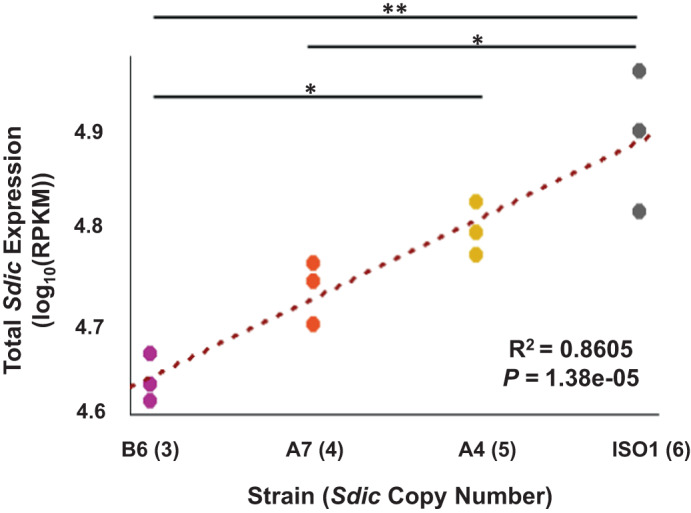
Total *Sdic* expression in testis and *Sdic* CN are positively correlated. Linear regression plot between *Sdic* CN and *Sdic* expression when all paralogs are surveyed jointly in each strain. The coefficient of determination (*R*^2^) and its corresponding *P* value are indicated. Black bars connect significant pairwise tests (Tukey HSD) for differences in expression levels; *P < 0.05; **P < 0.01. Statistical values for all comparisons are listed in Supplementary Table 4. Normalized expression values (*n* = 3 biological) are provided as log_10_(RPKM) and color-coded differently for each strain (Supplementary Data 2).

We detected consistent *Sdic* expression across replicates and strains (Supplementary Fig. 3). Total *Sdic* expression was found to be positively correlated with CN (Fig. 2; *R*^2^ = 0.8605, *P* = 1.38e–05). This finding is reflected in the significant differences in mRNA abundance detected (Fig. 2 and Supplementary Table 4; *P* = 7.52e–04, one-way ANOVA), although in an imperfect manner as pairs of strains differing by a single copy, e.g., between the strains ISO1 and A4 (6 vs 5 copies), do not show significant differences in mRNA abundance (Supplementary Table 4; *P* = 0.108, pairwise Tukey HSD), denoting some degree of buffering. Overall, these results challenge the previous inference, based on whole-body data, that variation in expression modifiers acting primarily in *trans* was the main factor shaping *Sdic* naturally occurring expression variation^25^ while highlighting the importance of tissue-level surveys of gene expression to prevent distorted accounts of biologically relevant expression patterns^32^. Further, our findings suggest an absence of regulatory mechanisms maintaining a stable total *Sdic* expression level in testis despite variation in *Sdic* CN. This is compatible with the *Sdic* expression level being under positive selection, possibly in connection with *Sdic*’s impact on sperm competitive ability^28^, at least in the CN range studied here. This is particularly relevant as the Sdic protein might be part of a protein complex, and expression level modifications as a result of CN changes for this type of gene product are presumably under stronger purifying selection due to stoichiometric constraints^22,42^.

### Vast evidence of intrastrain divergent expression among *Sdic* paralogs in testis

Despite their young age, *Sdic* paralogs have accumulated some nucleotide changes in the coding sequence, promoters, and UTRs^25,27^. These nucleotide differences can influence mRNA abundance pre- and post-transcriptionally^49^. In fact, early assessments of publicly available RNA-seq data suggested some degree of paralog expression profile differentiation^27^. However, technical constraints in many of the RNA-seq datasets analyzed, e.g., unstranded libraries or limited sequencing depth, reduced the reliability of the inferences made.

**Fig. 3:**
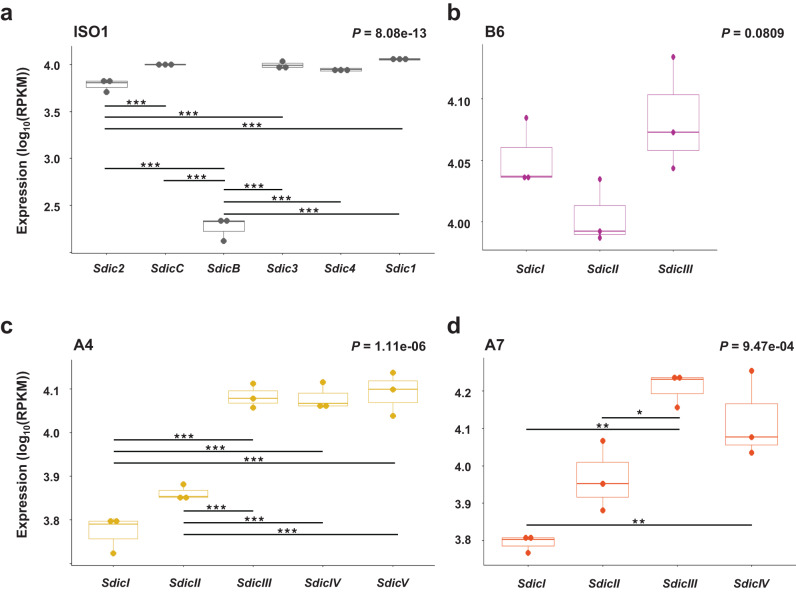
Individual *Sdic* paralogs show significant expression differences within strains. Expression of the individual *Sdic* paralogs in ISO1 (**a**), B6 (**b**), A4 (**c**), and A7 (**d**) is provided as log_10_(RPKM). Strains are color-coded as in Fig. 2. Boxes represent the interquartile range (IQR) around the median (horizontal black line) and whiskers extend to 1.5 times the IQR. *Sdic* paralogs are shown from left to right as they are arranged along the cluster from centromere to telomere in each strain^25^. In the reference strain ISO1, *SdicB* is significantly less expressed than the other paralogs, and *Sdic2* is significantly lower expressed than *SdicC*, *Sdic3* and *Sdic1*. In A4, *SdicI* and *SdicII* show significantly decreased expression relative to the other paralogs but not to each other. In A7, the paralog that harbors a TE insertion, *SdicIII*, has the highest transcript abundance, although it is only significantly greater than that of *SdicI* and *SdicII*. In this strain, *Sdic1*-like, i.e. *SdicIV*, is only significantly more expressed than *SdicI*. One-way ANOVA *P* values are indicated. Black bars connect significant pairwise comparisons (Tukey HSD); *P < 0.05; **P < 0.01; ***P < 0.001. Statistical values for all comparisons are listed in Supplementary Table 5 and normalized expression values (*n* = 3 biological) per paralog and strain are provided in Supplementary Data 3.

Leveraging on the precise knowledge of the nucleotide differences among *Sdic* paralogs, we tracked their individual testis expression across the strains of panel I using the same computational pipeline as above (Supplementary Figs. 1 and 4 and Supplementary Table 3). Upon confirming the expression of all paralogs regardless of the strain, we scrutinized intrastrain significant differences among paralogs. Except for ISO1, in which we follow an upgraded notation of that in FlyBase^27^, the paralogs in the remaining strains were numbered from I (downstream *sw*) to *n* (upstream *AnxB10*). The single paralog that is present across strains is the one adjacent to *AnxB10*, the one that corresponds to *Sdic1* in ISO1, *Sdic1*-like hereafter for other strains. In all strains except for B6 (*P* = 0.0809; one-way ANOVA), we documented significant differences in expression: ISO1, *P* = 8.08e–13; A4, *P* = 1.11e–06; and A7, *P* = 9.47e–04 (Fig. 3 and Supplementary Table 5; one-way ANOVA). For one case, *SdicB* in ISO1, the low expression detected could be partly explained by the fact that the motif sequence used to track expression overlapped with the very end of the 3’UTR, and therefore could be more impacted by exosome degradation^50^. Further, and upon omitting *SdicB* in ISO1, expression differences between the most and least expressed paralogs ranged from 56% in B6 to 240% in A7. Particularly noticeable is the case of the enhanced expression of a paralog with a premature stop codon induced by a 17.5 kb TE insertion in the fourth exon of *SdicIII* in A7 (Supplementary Fig. 5). Sequence analysis of its open reading frame indicated that it still has the potential to generate a functional Sdic protein^25^. The elevated expression of this paralog challenges the common perception of TE insertions largely exerting downregulatory effects^51,52^.

Expression differences do not explicitly inform about significant departures from an equal partitioning of the total *Sdic* expression level among paralogs. We performed Monte Carlo simulations (Methods) for each of the strains to determine the extent to which the average contribution of any individual paralog to the total expression in a given strain was significantly higher or lower relative to that expected under an equal partitioning of the total expression level (*P*_adj_ < 0.05; Supplementary Fig. 6, Supplementary Table 6, and Supplementary Data 4). While most paralogs were found to be expressed in a non-significantly different manner from the expected level according to an equal contribution to total *Sdic* expression, three paralogs were found to contribute significantly less (at *P*_adj_ < 0.05; *SdicB* in ISO1, *SdicI* in A4, and *SdicI* in A7) and one more (at *P*_adj_ < 0.05; *Sdic1* in ISO1) than such presumed equal contribution. As mentioned above, the pattern associated with *SdicB* could be artifactual.

Beyond the possibly up-regulatory TE-mediated effect detected in A7, nucleotides changes in promoter sequences could also contribute to paralog differentiation at the expression level. Sequence alignments revealed just two nucleotide sites being affected, producing four promoter types (Fig. 1b). A joint examination of promoter diversity and expression levels among paralogs (Figs. 1b and 3) did not reveal any robust association. For example, in ISO1, *Sdic2*, *SdicC*, *Sdic4*, and *Sdic1* have exactly the same promoter type but are expressed differentially, with *Sdic*2 being expressed at a significantly lower level. In A7, all paralogs possess the same promoter, which does not prevent expression differences among paralogs. In all strains, the only paralog in common (*Sdic1/Sdic1-*like) shows either the highest expression level or no difference in expression relative to the most highly expressed paralog. This paralog displays different promoters across strains, which could result from de novo mutations or gene conversion, the latter being known to be rampant in the *Sdic* region^25,27^. Lastly, beyond B6 and upon omitting *SdicB* from ISO1, we found no unequivocal evidence that the relative position of an *Sdic* copy within the tandem array impacts its expression level in any particular direction, i.e. toward overall higher or lower expression levels. Nevertheless, the paralog adjacent to *sw* exhibited downregulation in relation to many others in ISO1, A4, and A7.

### Cell-specific expression data reveal differences in spatial regulation among *Sdic* paralogs

Paralog differentiation can also take place spatially within the testis as early analyses on scRNA-seq data from adult testis suggested^53,54^. Nevertheless, such analyses used an outdated gene annotation for the *Sdic* region when interpreting read mapping results and did not consider pre-eclosion stages, when *Sdic* expression has already started^55^. Leveraging an upgraded annotation of the *Sdic* region in the reference strain^27^, scRNA-seq data from third-instar (L3) larva testis^33^, snRNA-seq data from 0-1 d old adult testis^34^, and a common computational pipeline (Methods), we investigated the differentiation of expression profiles among paralogs across cell types while considering two different time points of male development. Through unsupervised clustering, we delineated 12 unique clusters in L3 testis, and adopted 36 previously annotated clusters in Fly Cell Atlas for adult testis, respectively. These clusters were annotated within the somatic and germline cell categories (Supplementary Figs. 7 and 8 and Supplementary Table 7), using already well-established marker genes for different cell types^33,34^.

**Fig. 4:**
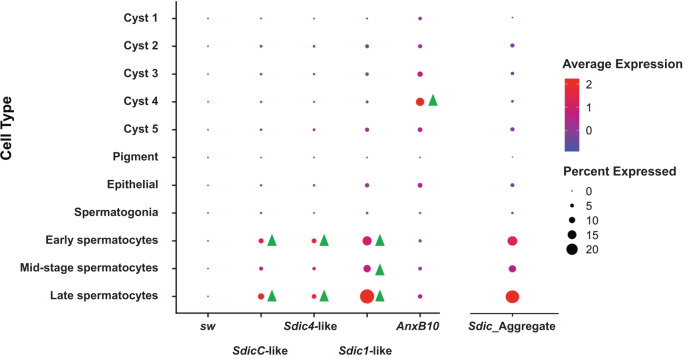
Differential expression of *Sdic* paralogs across testis cell populations at the third-instar larva of *D. melanogaster*. Cell types are indicated on the *y*-axis and the different *Sdic* paralogs appear on the *x*-axis. The flanking parental genes *sw* and *AnxB10* are included for the sake of completeness. The order of the genes (from left to right) mirrors that in the genome (from centromere to telomere) of ISO1. This order does not necessarily need to be the same in *w*^*1118*^, a strain in which the *Sdic* region has not been resolved. The aggregate expression level considering all *Sdic* copies for which we find evidence of expression is additionally shown. Average expression is color-coded, reflecting the z-scores calculated by scaling the log(corrected counts), which are in turn computed using the SCTransform v2 regularization. The diameter of the bubbles denotes the percentage of cells within a given cluster that expresses the respective gene. Green triangles indicate cell types for which a particular paralog showed significantly higher expression relative to the average expression level in the rest of the cell types (*P*_adj_ < 0.05). The paralogs *SdicB-*like and *Sdic3-*like are not represented as no detectable expression of them was found. Normalized expression levels are provided in Supplementary Data 5.

Apart from *Sdic3-*like, all other paralogs were found to be expressed in L3 larva or adult; ~9.7% and ~19.5% of the cells express *Sdic* in L3 larvae and adults, respectively (Fig. 4, Supplementary Fig. 9, and Supplementary Data 5). As the data used belongs to the strain *w*^*1118*^ and not to the reference strain ISO1, i.e., the strain whose genome assembly is used in the read mapping process, the lack of expression of *Sdic3-*like could represent a genuine low or absent expression, or a technical limitation if this paralog does not exist as such in *w*^*1118*^. Likewise, the detection of expression for *SdicB-*like and *Sdic2-*like in adult but not in L3 larva could reflect bona fide developmental regulation or merely a lower sequencing depth in L3 larva, which is in fact the case (Supplementary Table 8). At both stages, *Sdic1-*like exhibits the highest expression followed by *SdicC-*like, which is compatible with the existence of expression differences among *Sdic* paralogs in *w*^*1118*^.

In the context of the progression of spermatogenesis, *Sdic1-*like*, Sdic4-*like, and *SdicC-*like exhibit increasingly higher expression as spermatogenesis progresses in L3 larva, peaking primarily in late spermatocytes. In adults, the pattern is similar, with preferential expression in late spermatocyte stages, which also includes *SdicB-*like and *Sdic2-*like, and subsequently with sustained expression in spermatids. Regardless of the differences among paralogs, their expression patterns are positively correlated across cell types at both developmental stages (Supplementary Table 9). Further, the aggregate expression across paralogs during spermatogenesis faithfully recapitulates the indicated global patterns (Fig. 4 and Supplementary Fig. 9). Crucially as well, when using the motif-counter approach described above, which is a reliable but conservative approach that does not require any mapping against the reference strain ISO1, we confirmed the same trends (Supplementary Data 5). Collectively, these results confirm a preferential expression of the *Sdic* multigene family late during spermatogenesis at the two developmental stages examined.

Our results also indicate that the expression of *Sdic* paralogs is more tightly associated with meiotic than with somatic or mitotic (spermatogonia) cell types. This aligns well with the fact that meiotic cells are characterized by a more favorable molecular environment that facilitates the expression of newly evolved genes^56–58^, with a putatively preferential role of the Sdic protein in the accumulation or maturation of spermatocytes^55^, or both. It must be noted that unlike previous reports in adult testis^54^ we did find evidence of *Sdic* expression in spermatogonia. This discrepancy may arise from the increased granularity in cell clustering implemented here^34^.

Cell- and nucleus-level expression differences among *Sdic* paralogs are intriguing. The *X* chromosome becomes increasingly inactivated as spermatogenesis progresses, consequently downregulating *X-*linked genes, which become less accessible to the transcriptional machinery^33,59^. Coinciding with others, although using an outdated annotation of the *Sdic* cluster^54^, particularly the paralogs *Sdic1-*like*, SdicC-*like, and *Sdic4-*like appear to escape the effects of this epigenetic mechanism. It is unclear at this time what mechanism gives rise to this diversity of paralog expression patterns among testis cell types at the L3 and adult stages. Future paralog-focused expression surveys for *Sdic* and other young multigene families should leverage strain-specific annotation and sequence information to delineate in the most reliable manner true differences in paralog expression across developmental stages.

### The *Y* chromosome differentially impacts *Sdic* expression across male reproductive tissues

*Y*-linked regulatory variation (YRV) has been shown to impact the expression of 20–40% of genes expressed in testis and, to a lesser extent, in somatic tissues of *D. melanogaster*^35,60^. This *trans*-regulatory effect is very dynamic even within species^61^, possibly reflecting the fast evolution of the TE content and other repeat-related loci on the *Y* chromosome^62,63^. We hypothesized that the *Y* chromosome could act as a *trans*-regulator of *Sdic* expression. As no previous analysis on the tissue-level impact of YRV has been conducted using an analytical pipeline dedicated to distinguishing expression between genes extremely similar in their nucleotide sequences, e.g., *Sdic* and its parental gene *sw*, we tested the effect of different *Y* chromosomes on *Sdic* expression within a common genetic background, specifically that of the strain 4361, following others^35^.

We generated six *Y* chromosome substitution lines following a previously established mating scheme^35^ (Panel II in Supplementary Table 1 and Fig. 1a) on which we performed two different expression analyses. First, we assayed total *Sdic* and *Sdic1*-like expression in male whole bodies using qRT–PCR (Supplementary Fig. 10 and Supplementary Data 6). For that, we used primer sets that target a region in the first exon conserved across all *Sdic* paralogs and a region in the last exon only conserved across all *Sdic1*-like paralogs, respectively. We found significant differences in total *Sdic* expression (Supplementary Fig. 10a; *P* = 0.0153, one-way ANOVA; Supplementary Table 10), with A7y and B3y showing higher expression than ORRy (*P* < 0.05 in both cases; Tukey HSD). No difference in expression was found for *Sdic1-*like alone (Supplementary Fig. 10b, *P* = 0.291, one-way ANOVA; Supplementary Table 10). This result suggests that the *Y* chromosome has a regulatory impact on the expression of the *Sdic* multigene family that contributes to interstrain expression differences, although this effect does not necessarily affect each *Sdic* paralog.

**Fig. 5:**
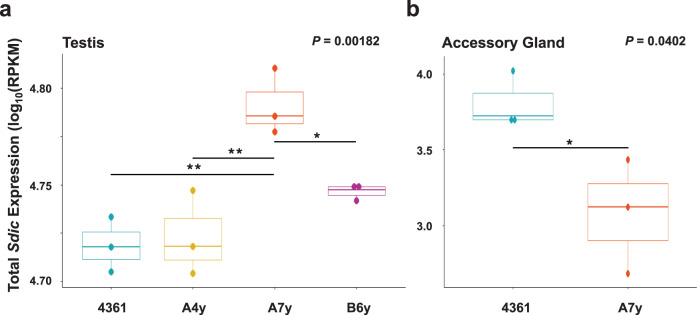
*Y*-linked regulatory variation differentially impacts total *Sdic* expression in the testis and accessory gland. One-way ANOVAs comparing total *Sdic* expression in testis (**a**) and male accessory gland (**b**) across strains of panel II. *P *values are indicated. Normalized expression is plotted as log_10_(RPKM). Boxes represent the interquartile range (IQR) around the median (horizontal black line) and whiskers extend to 1.5 times the IQR. Black bars connect significant pairwise comparisons (Tukey HSD). *P < 0.05; **P < 0.01. Statistical values for all comparisons are listed in Supplementary Table 11 and normalized expression values (*n* = 3 biological) per strain are provided in Supplementary Data 7.

Subsequently, we performed RNA-sequencing at the tissue level to better detect any biologically relevant regulatory effect of the *Y* chromosome on total *Sdic* expression (Supplementary Data 7). We did so in the testis across four strains (4361, A4y, A7y, B6y) and male accessory gland across two strains (4361, A7y). Total *Sdic* expression in testis significantly differs across the *Y* chromosome substitution panel, being particularly increased in A7y (19.4% more than in 4361; Fig. 5a and Supplementary Table 11; *P* = 0.0018, one-way ANOVA). Notably, the *Y* chromosome also has a differential impact on the total *Sdic* expression in male accessory gland, but with A7y showing significantly decreased expression compared to 4361 (406% less than in 4361; Fig. 5b and Supplementary Table 11; *P* = 0.0402, one-way ANOVA).

The *trans*-regulatory effect of YRV on *Sdic* expression both in the germline and in a somatic reproductive tissue (accessory gland) is important for several reasons. First, *Sdic* expression level was upregulated in the testis but downregulated in the accessory gland of A7y relative to 4361, demonstrating potentially opposing regulatory effects of YRV on *X*-linked genes across different tissues of the male reproductive system. The relevance of this result should be taken cautiously as it involves only two strains. Nevertheless, it can be the tip of a more complex landscape of regulatory effects across tissues, sometimes agonistic, sometimes antagonistic, that should be explored across multiple genotypes. Second, our results show how the *Y* chromosome not only can impact sperm competitiveness—and therefore male fertility—by harboring sperm axonemal motor protein-encoding genes^64^, but also through the regulation of novel genetic factors elsewhere in the genome such as species-specific genes and therefore regardless of their evolutionary age^28,29^.

### Conclusions

Here we provide a nuanced portrait of how quickly a recently formed tandem gene family has become diversified for several expression attributes while becoming a stable component of the gene repertoire of *D. melanogaster. Sdic* shows evidence of asymmetrical partitioning of its total expression level among paralogs, and its total expression is affected by both CNV and *Y* chromosome origin, the latter showing some evidence of opposed regulatory effects across male reproductive tissues. Future studies using genetically engineered lines will be necessary to understand how specific genetic changes in promoters, 3’UTRs, and other *cis-*regulatory modules impact the expression of individual paralogs. Further, we ignore whether differences in mRNA abundance among paralogs are maintained at the protein level or are stabilized through post-transcriptional or -translational buffering mechanisms. Precise quantification of the level of Sdic protein at the tissue-, or ideally cellular-, level will clarify this end.

## Methods

### Drosophila husbandry

We used the following *D. melanogaster* strains: the reference strain ISO1 and three wild-type strains with diverse geographical origin (panel I)^65^; 4361 (Bloomington Stock Center); and six *Y* chromosome substitution lines (panel II) (Supplementary Table 1). Flies were reared on dextrose-cornmeal-yeast medium at room temperature (~25 °C) under 24 h fluorescent light. Adult virgins were collected within 6–8 h of eclosion, sorted by sex, and then cultured in groups of ≤50 individuals until sacrificed. All manipulation of flies was performed under CO_2_ anesthesia.

### Generation of *Y* chromosome substitution lines

Crosses to generate the *Y* chromosome substitution lines (panel II; Supplementary Table 1) were done following a previously described mating scheme^35^. Briefly, the *D. melanogaster* strain 4361 is used as it carries a recessive marker in each of the four chromosomes. Females from this strain were crossed with males carrying *Y* chromosomes reflecting a wide variation in origin. The resulting F1 males were then backcrossed with 4361 females such that the F2 males, those to be expression profiled, had an identical genomic background to that of 4361 except for the *Y* chromosome, which derives from a particular donor strain.

### Tissue dissections

For panel I, 5 d post-eclosion naive males had their testis dissected. For panel II, a similar approach was followed with testis and male accessory gland of 4-6 d post-eclosion males. Tissue dissection was performed in 1×PBS (phosphate-buffered saline) solution and stored in ice-cold 1×PBS for less than 2 h. Following dissection, 1×PBS was replaced with TRIzol (ThermoFisher). Tissues were homogenized using a 1.5 mL motorized pestle, flash-frozen in liquid nitrogen, and immediately transferred to a −80 °C freezer until used for RNA extractions. Dissections were done separately for each strain, tissue, and sex to avoid possible cross-contamination. Tissues were dissected within specific timeframes to minimize unintended variation.

### RNA extractions

Immediately prior to RNA extraction, tissues previously homogenized in TRIzol were pooled: 25 male whole bodies; 100 pairs of accessory glands; and 60 and 100 pairs of testes for the panels of strains I and II, respectively. Four replicates were extracted for each sample type. Total RNA was extracted using chloroform following manufacturer instructions for TRIzol. DNA traces were eliminated using the RNeasy mini kit with DNase I (Qiagen). RNA integrity, purity, and concentration were assessed by gel electrophoresis, a Nanodrop-8000 spectrophotometer (ThermoFisher), and a Qubit RNA BR assay kit (ThermoFisher), respectively. Extracted total RNA was immediately stored at −80 °C until used for cDNA synthesis or submitted for sequencing.

### qRT–PCR analysis

We followed a previous protocol^25^. Briefly, four biological replicates were quantified per strain. Expression estimates were obtained accounting for variable primer efficiencies for the amplicons of interest (*Sdic*_All, *Sdic1*-like) and the reference gene *clot* (*cl*)^66^. Samples from strain 4361 were used as the calibrator for all comparisons. Primers and conditions implemented are given in Supplementary Table 12. Primer design for *Sdic* took into consideration sequence similarities and differences with *sw* and *AnxB10* to confidently survey solely *Sdic* expression. To estimate the combined expression of all *Sdic* paralogs, the *Sdic_*All primers target a region with perfect sequence conservation across all paralogs and strains, which prevents any paralog or strain bias. Likewise, the priming sites for *Sdic1*-like target a region that is conserved across all *Sdic1*-like paralogs reliably annotated, and none of the other paralogs in this study. All samples tested with the same primer set were run on the same 96-well plate.

### RNA-sequencing

Samples from the panels of strains I and II were sequenced separately. Prior to sequencing, RNA integrity was estimated using the RNA 6000 Nano Chip Kit (Agilent Technologies) with an Agilent 2100 Bioanalyzer. For each sample, the three out of the four replicates with the highest RIN values were submitted for RNA-sequencing at the UCI Genomics Research and Technology Hub (GRT Hub). Ribodepleted, strand-specific paired-end libraries were prepared according to the Illumina TruSeq Total RNA stranded protocol. The resulting libraries were validated by qPCR and sized by Agilent Bioanalyzer DNA high-sensitivity chip. Library concentrations were normalized and then multiplexed together. The multiplexed libraries were sequenced using paired-end 100 cycles chemistry on a NovaSeq 6000 instrument.

### RNA-seq data processing and gene expression quantification

Whether generated as part of this work or elsewhere^67–69^, quality control and pre-processing of RNA-seq reads were performed using HTStream (https://github.com/s4hts/HTStream; last accessed February 14, 2022), including removal of known *D. melanogaster* rRNA-related sequences as presented in NCBI, adapter sequences, reads shorter than 50 nt, and filtered for low-quality bases using a sliding window approach^70^. The RNA-seq libraries generated elsewhere correspond to those from Leader et al.^67^ (ERR2103700, ERR2103701, ERR2103705, ERR2103706, ERR2103707, ERR2105061, ERR2105062, ERR2105063, ERR2105064, ERR2105065, ERR2105066, ERR2196289, ERR2196290, ERR2196291, ERR2196292, ERR2617942, ERR2617943, ERR2617951, ERR2617952, ERR2103702, ERR2103703, ERR2103704, ERR2196293, ERR2196294, ERR2098815, ERR2098816, ERR2098817, ERR2098818, ERR2098819, ERR2098820, ERR2103041, ERR2103042, ERR2103043, ERR2103038, ERR2103039, ERR2103040, ERR2617868, ERR2617869, ERR2617949, ERR2617950, ERR2099027, ERR2099028, ERR2099029, ERR2196295, ERR2196296, ERR2196297, ERR2102258, ERR2102259, ERR2102260, ERR2105722, ERR2105723, ERR2105724, ERR2107425, ERR2107426, ERR2107427), Brown et al.^69^ (SRR023607, SRR029230, SRR029234, SRR023543, SRR035394, SRR023601, SRR035395, SRR023605, SRR023606, SRR029176, SRR029231, SRR029233, SRR029235, SRR023602, SRR024012, SRR035399, SRR023540, SRR035400, SRR023600, SRR035402, SRR023599, SRR027114, SRR035403, SRR023506, SRR027109, SRR023538, SRR024015, SRR023604, SRR024010, SRR035397, SRR023502, SRR027112, SRR023539, SRR035405, SRR035406, SRR023504, SRR027113, SRR035407, SRR023596, SRR023603, SRR035409, SRR026431, SRR023199, SRR027110, SRR035417, SRR023597, SRR035410, SRR023542, SRR035412, SRR023507, SRR027111, SRR026433, SRR023546, SRR023608, SRR035413, SRR023505, SRR027108, SRR023544, SRR035414, SRR023541, SRR035415, SRR026430, SRR035391, SRR023609, SRR035416), and Chen et al.^68^ (SRR1712836, SRR1711806). Gene expression was quantified for the entire *Sdic* multigene gene family and for each individual paralog by using a motif-counter pipeline^27,71^, which detects and counts sequencing reads using a custom script that scrutinizes each library for the presence of a given motif. Briefly, this pipeline first searches for reads with perfect matches to a 20-nt core motif unique to either an individual *Sdic* paralog or, in the case of measuring total *Sdic* expression level, the first exon of *Sdic* which is conserved across all *Sdic* paralogs (Supplementary Fig. 1b). Then, the pipeline screens those reads to identify those with ≤1 mismatch to a 130-nt extended motif that extends 55 nt to each side of the core motif (Supplementary Fig. 1a; Supplementary Table 3). Normalized counts are expressed as reads per kilobase of transcript per million mapped reads (RPKM)^72^. It must be noted that this normalization was done considering the number of reads uniquely mapped to a particular motif relative to the total number of reads in the fastq files and not, as is common practice, to the number of mapped reads against a genome assembly as no such procedure is implemented in the motif-counter pipeline^27^. Further, in our implementation the variable length is irrelevant as all the motifs are 130-nt long^27^. A minimum of ten reads in all three replicates from the same tissue and strain was used as a threshold for dubbing expression as reliable and meaningful biologically.

### Statistics and reproducibility

One-way ANOVA was implemented to test for differences in mRNA abundance among strains or within strains. Expression values were log_10_ transformed. Homogeneity of variance and normality were tested with the Levene and Shapiro–Wilk tests, respectively^73^. For tests with statistically significant *P* values, post-hoc Tukey–Kramer HSD tests were performed to identify statistically significant pairwise comparisons while correcting for multiple tests. Gene expression correlations were calculated using Pearson’s correlation coefficient, and the resulting *P* values corrected for multiple tests^74^. All these statistical analyses were performed in R^75^. For the paralog partitioning expression analysis, Monte Carlo simulations were performed for each strain on the expression values from the individual replicates and paralogs. Expression values for each strain were permutated 10,000 times to calculate the probability of finding an average expression per paralog higher or lower than presumed assuming an even contribution from all paralogs, with such probabilities being corrected for multiple tests^74^. A significantly higher or lower contribution was set at *P*_adj_ < 0.05.

### Sc- and snRNA-seq analysis

Two previously published sc- and snRNA-seq datasets^33,53^ from *D. melanogaster* third-instar (L3) larva and 0–1-d-old adult testis, were retrieved from NCBI and EBI, respectively. The datasets represent biological replicates from larva (SRR8513906, SRR8513907, SRR8513908) and adult (ERS6860763, ERS6860767, ERS6860771) testis from individuals of the strain *w*^*1118*^. For the L3 dataset, the *mkref* command of CellRanger v3.1.0^76^ was used to create a reference transcriptome based on release 6.32 of the annotation of *D. melanogaster*. The paired-end reads were then aligned to this reference transcriptome for the strain ISO1^77^ using the CellRanger command *count*, ultimately allowing to demultiplex the single-cell sequencing reads into a usable format for Seurat. The raw counts for the adult testis dataset were obtained from the r_fca_biohub_testis_10x.loom file, which was converted to a Seurat object for downstream processing. On Seurat v4.0.5^78^, visualization tools were used to gauge the median number of cells and genes expressed in each pre-filtered object. Genes expressed in at least three cells and all cells with 200–8000 genes expressed were retained for downstream analyses. Using this normalized dataset, DoubletFinder v.2.3^79^ was applied to remove doublets from the L3 dataset following log normalization. Only pre-annotated cells were kept for downstream processing in the case of the adult dataset, thus making doublet removal from this dataset redundant. Subsequently, the data across all samples were normalized using the SCTransform v2 command under default parameters. At this stage, in the case of the larva data, the results from the three samples were combined into a single Seurat object. The default UMAP function (RunPCA, RunUMAP, FindNeighbors, FindClusters) was then used for unsupervised clustering of the combined larva Seurat object. Clusters were identified based on the first 10 principal components (resolution = 0.5). Testing different numbers of principal components did not result in substantially different clustering patterns, but 10 principal components generated the best separation between different cell types for both datasets. Cluster identification was done using known marker genes (Supplementary Table 7)^33^. Some pairs of cell clusters were collapsed into one based on the expression patterns of the marker genes while others remained unidentified as the expression profiles of these clusters were inconclusive for key marker genes. For the adult dataset, the original annotation from Fly Cell Atlas was retained, thereby removing the need for manual cluster identification using marker genes. For both datasets, the output for each paralog included the normalized expression level per cell type and whether the expression in each cell type was significantly different from the rest of cell types, which was determined using Wilcoxon rank sum tests (one per cell type) and by correcting the resulting *P* values for multiple tests^74^. Subsequently, combining the normalized expression values per cell type and corrected *P* values helped determine in what specific cell types a given paralog was significantly peaking in expression and in which cell type in particular its expression was the highest. Lastly, the aggregate expression of all *Sdic* paralogs was calculated by adding their counts and plotting the average expression across the different cell types for both the L3 and adult datasets.

In an independent approach to quantify *Sdic* expression across cell types, the raw reads from both datasets were also subject to our motif-counter pipeline after pre-processing using HTStream. The core and extended sequence motifs for *Sdic*_All (common to all *Sdic* paralogs characterized to date) and *Sdic1/Sdic1*-like (the only *Sdic* paralog present in all strains analyzed to date) implemented in the intrastrain analysis were used to count the number of reads containing perfect matches. These reads were then annotated for their cell type using a custom Python script based on our previous cell cluster annotation, and normalized counts were expressed as RPKM.

### Reporting summary

Further information on research design is available in the Nature Portfolio Reporting Summary linked to this article.

### Supplementary information

Supplementary Information

Description of Additional Supplementary Files

Supplementary Data 1-7

Reporting Summary

## Data Availability

Supplementary Data 1–7 include the source data behind Figs. 2–5 and Supplementary Figs. 2, 6, 9, 10. In the case of Supplementary Fig. 2, the libraries analyzed were obtained from refs. ^67^, ^69^, and ^68^. In the case of Fig. 4 and Supplementary Fig. 9, the libraries analyzed were obtained from ref. ^33^ and ref. ^53^, respectively. All these libraries are listed in “Methods”. All raw sequencing data generated in this work were deposited as part of the NCBI BioProject PRJNA971348.

## Code availability

The script of the previously published motif-counter pipeline^27^ is available at Zenodo^71^ (10.5281/zenodo.8408343).
