# Supplementary material for: Paralog transcriptional differentiation in the D. melanogaster-specific gene family Sdic across populations and spermatogenesis stages
Source: Commun Biol. 2023 Oct 20;6:1069. doi: 10.1038/s42003-023-05427-4 (PMC10589255; doi:10.1038/s42003-023-05427-4)
Supplement: Supplementary file 1 — Supplementary Information [file 42003_2023_5427_MOESM1_ESM.pdf]

SUPPLEMENTAL MATERIALS

(Contents: Supplementary tables 1-12; Supplementary figures 1-10)

Paralog transcriptional differentiation in the *D. melanogaster*-specific gene family *Sdic* across populations and spermatogenesis stages

Bryan D. Clifton [1] *, Imtiyaz Hariyani [1], Ashlyn Kimura [1], Sophia Luo [1], Alvin Nguyen [1], and José M. Ranz [1] *

[1] Department of Ecology and Evolutionary Biology, University of California Irvine, Irvine, CA 92697, USA

* Correspondence to bclifton@uci.edu and jranz@uci.edu

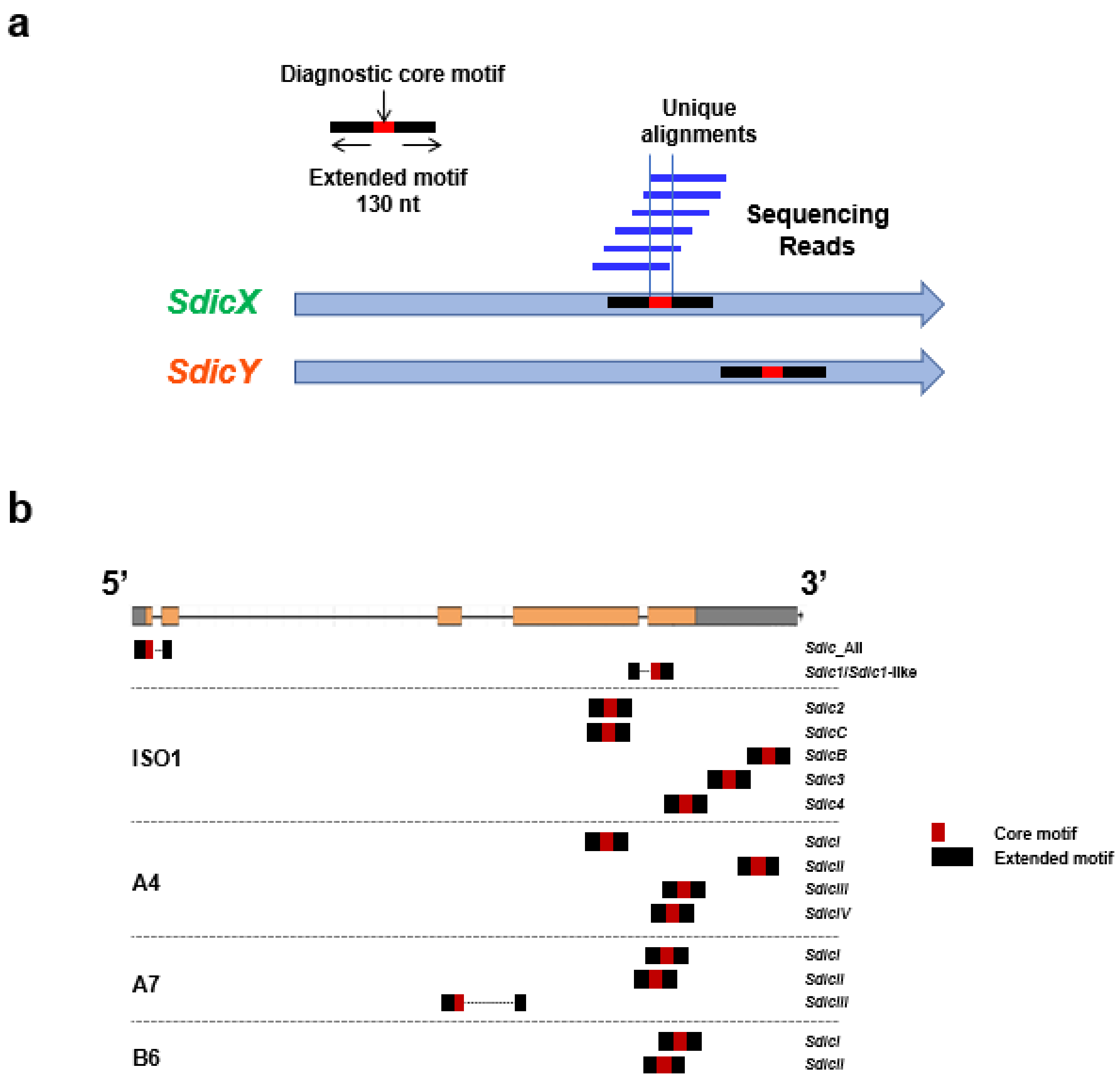

Supplementary Figure 1. Salient features of the motif-counter pipeline implemented to track *Sdic* expression. a In a given RNAseq library, the detection of sequencing reads that corresponds to a particular gene is performed by scrutinizing each library for the presence of a given motif. The pipeline first searches for reads with perfect matches to a 20 nt diagnostic core motif unique in this study to either an individual *Sdic* paralog or, in the case of measuring total *Sdic* expression level, the first exon of *Sdic* which is conserved across all *Sdic* paralogs. Such motifs are delineated wherever there are nucleotide differences unique to the gene of interest, which in this case varies in its location among paralogs. The pipeline then screens reads to identify those with $\leq$ 1 mismatch to a 130 nt extended motif that extends 55 nt to each side of the core motif. **b** Location of the sequence motifs used to quantify expression associated with *Sdic* paralogs across four strains of *D. melanogaster* (left). Both the core and the extended motifs used for monitoring the reads of each paralog, and in general for detecting all transcripts (*Sdic*_All), are indicated in relation to the exons and untranslated regions (orange and grey respectively) of a canonical *Sdic* copy. *Sdic1*/*Sdic*1-like, the only paralog present in all strains examined, is the only copy whose expression can be tracked using a common motif across strains.

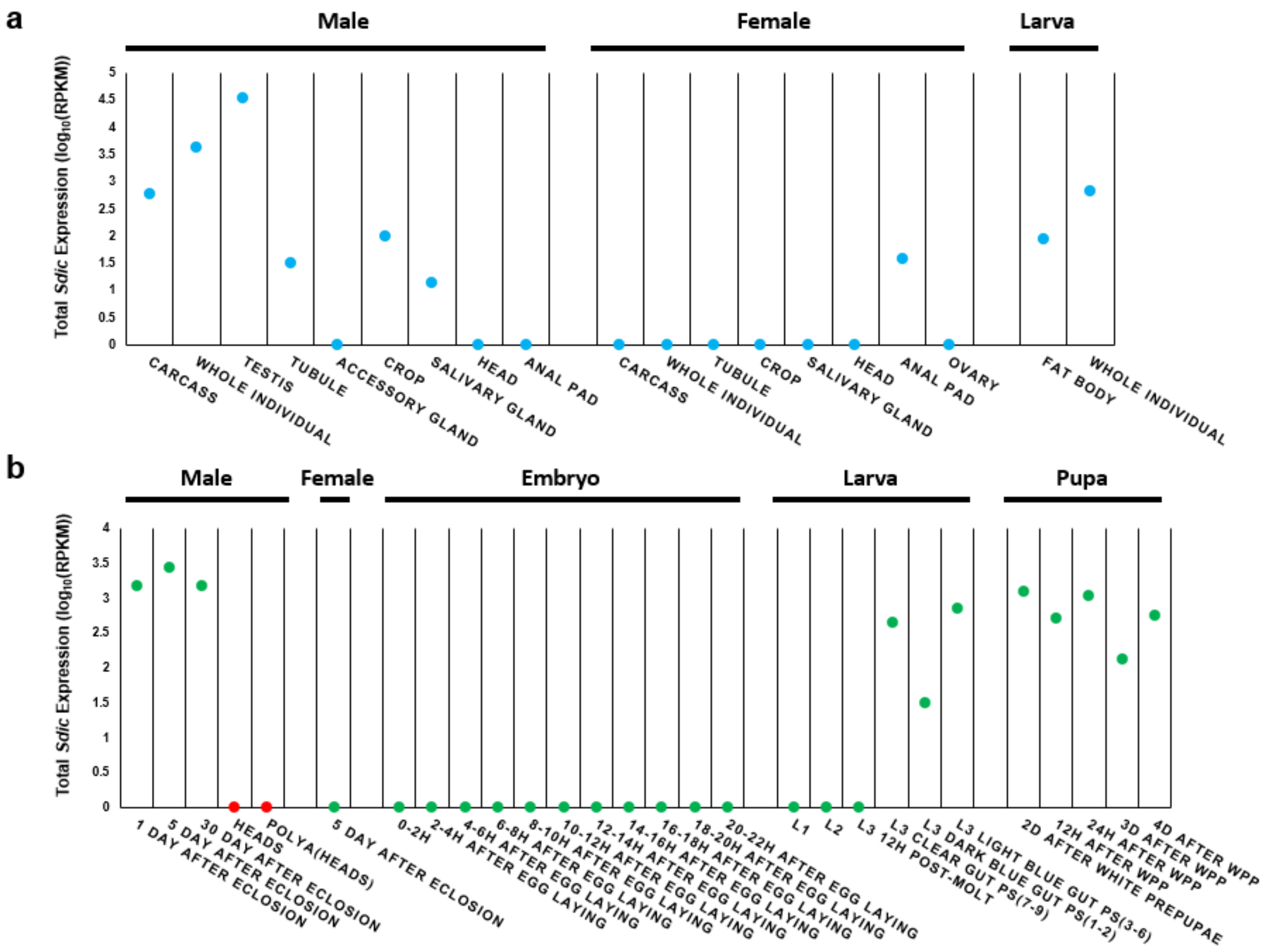

Supplementary Figure 2. Total *Sdic* expression across different samples. a Samples from FlyAtlas2 [1] are shown in blue. **b** Samples from Brown et al. and Chen et al. [2,3] are shown in green and red, respectively. All datasets used (55, 65, and 2, respectively) consisted of paired-end RNA sequencing reads. In the case that a particular sample type was represented by more than one biological replicate, the expression estimate corresponds to the average across biological replicates. The different datasets correspond to different tissues and body parts from adult and larva individuals, or to different whole-body organisms collected at different times during the life cycle. Normalized expression values are provided in Supplementary Data 1. The results support the preferential expression of *Sdic* in adult male samples, particularly in testis, or in mix-sexed samples of larva and pupa individuals. A conspicuous difference noticed in relation to previous conclusions derived from similar analyses using the same data [2] and pipeline is the absence of *Sdic* expression in heads of males and females, as well as in ovaries [4]. This is compatible with *Sdic* expression in heads and ovary being low and inconsistent, with too liberal parameter values being used in the past to run the computational pipeline [4], or both.

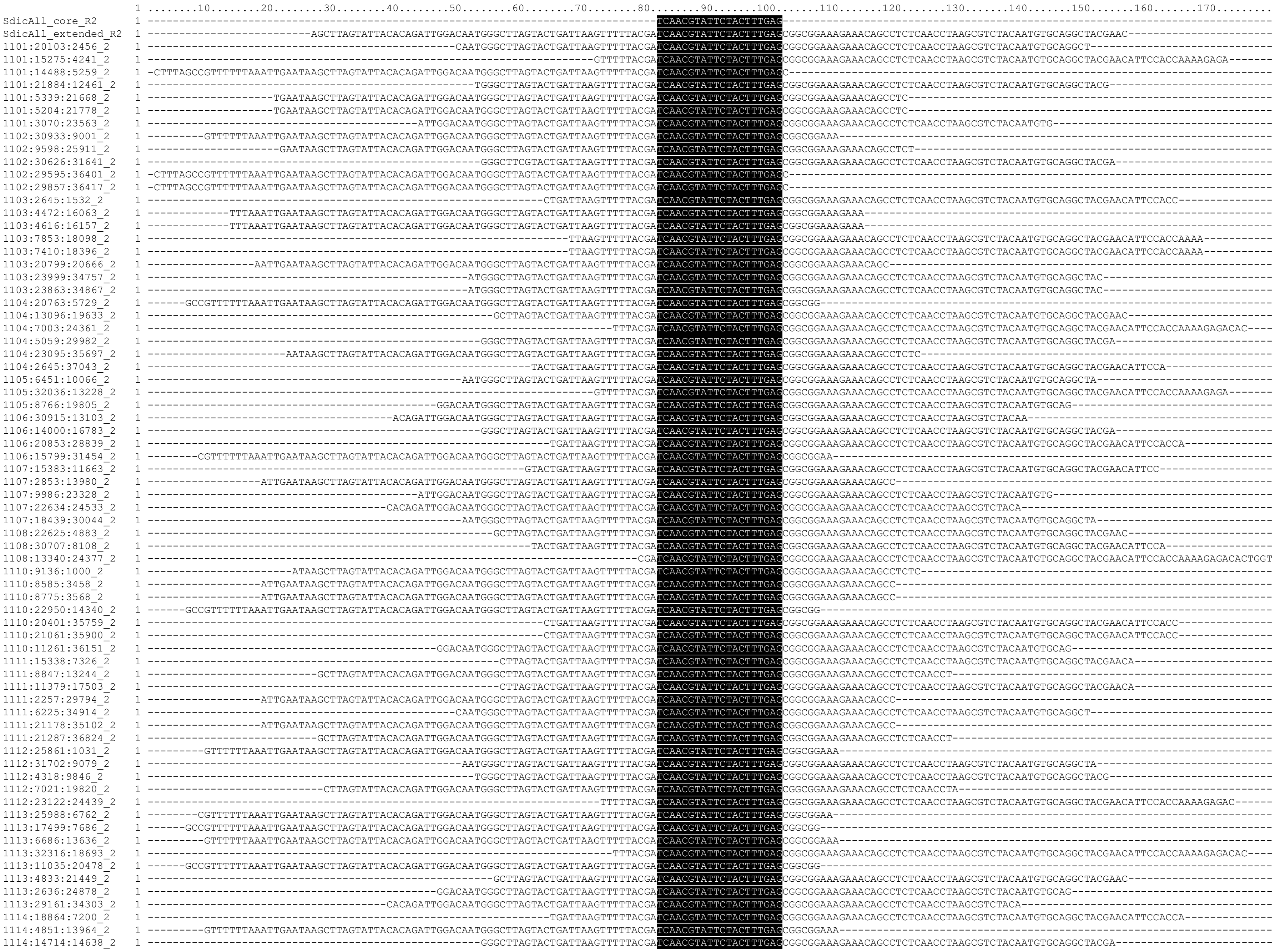

Supplementary Figure 3. Example of alignments of RNA-seq reads to a diagnostic motif that tracks the total *Sdic* expression in testis. RNA-seq reads with perfect alignments to 20 nt core motifs (shaded) and <u><</u>1 mismatch to 130 nt extended motifs (Supplementary Table 3) corresponding to total *Sdic* expression (*Sdic*_All) are shown. Sequences were aligned with MUSCLE in MEGAX [5], then shaded using Boxshade (https://github.com/mdbaron42/pyBoxshade). Only the first 100 reads detected from a single biological replicate of ISO1 are shown here. Reads with matches to both the sense and reverse complement sequences were counted, but only R2 alignments are shown here.

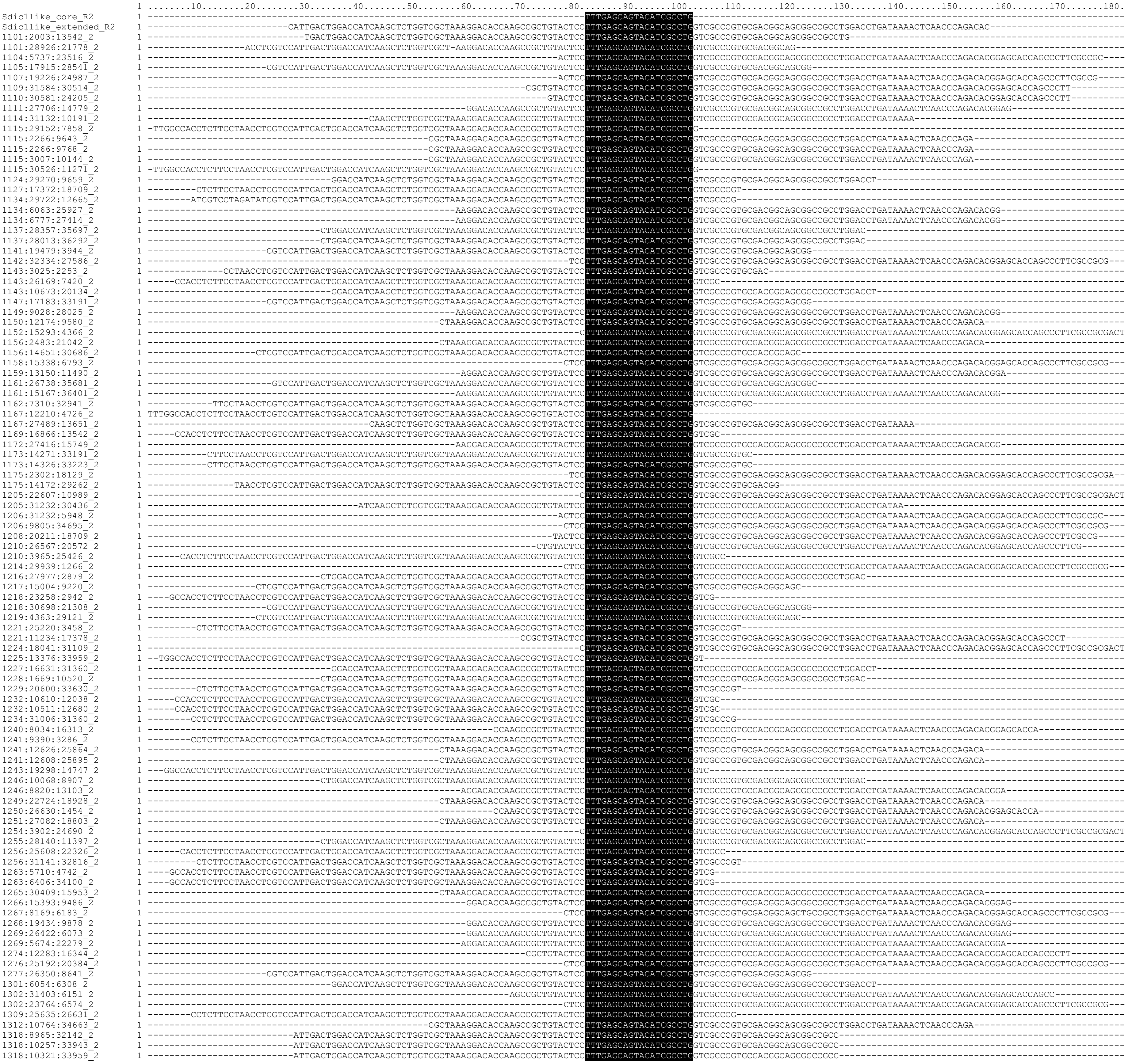

Supplementary Figure 4. Example of alignments of RNA-seq reads to a diagnostic motif that tracks the expression in testis of the only *Sdic* paralog present in all strains. RNA-seq reads with perfect alignments to 20 nt core motifs (shaded) and <u><</u>1 mismatch to 130 nt extended motifs (Supplementary Table 3) corresponding to the expression of the *Sdic* paralog present in all strains examined here (*Sdic1*-like) are shown. Sequences were aligned with MUSCLE in MEGAX [5], then shaded using Boxshade (https://github.com/mdbaron42/pyBoxshade). Only the first 100 reads detected from a single biological replicate of ISO1 are shown here. Reads with matches to both the sense and reverse complement sequences were counted, but only R2 alignments are shown here.

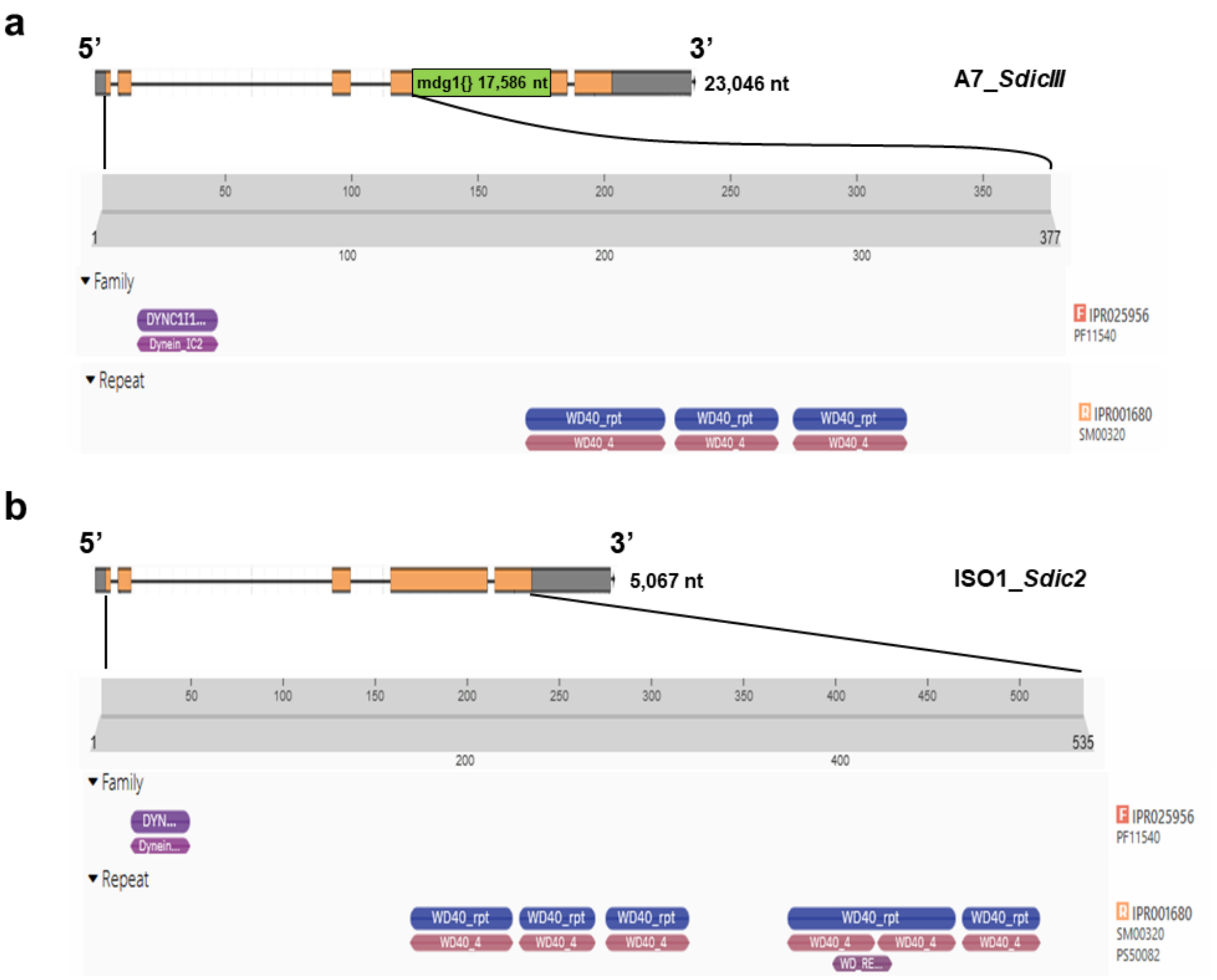

Supplementary Figure 5. Gene model and protein structured by the transposable element bearing paralog *SdicIII* in the strain A7. a Gene model of paralog *SdicIII* in strain A7. **b** Gene model of a similar paralog (*Sdic2* in the strain ISO1) shown for comparative purposes. The transposable element (*mdg1*) resides downstream of a *de novo* evolved STOP codon in *SdicIII*. Boxes, exons; lines, introns; orange, coding; grey, untranslated regions. The size of the TE is not to scale. Protein domain structure as annotated by InterPro 95.0 [6] are shown below the gene models. Total protein length in amino acids and corresponding coordinates of the protein domains are provided. The protein encoded by *SdicIII* is substantially shorter and carries fewer WD40 motifs than that encoded by other *Sdic* paralogs [7].

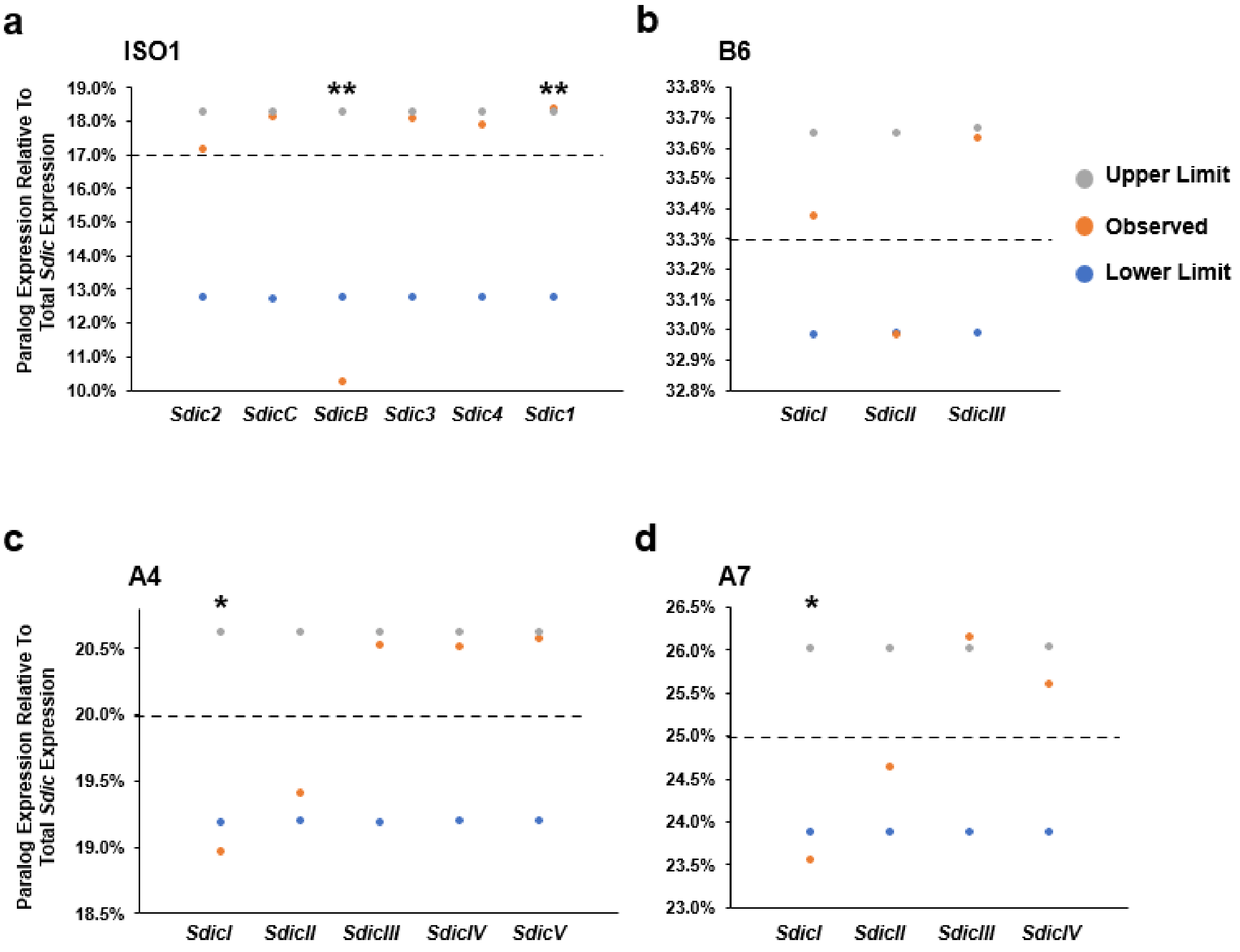

Supplementary Figure 6. Uneven contribution across paralogs to total *Sdic* expression. The expression of each individual *Sdic* paralogs in ISO1 (**a**), B6 (**b**), A4 (**c**), and A7 (**d**) relative to the total expression level of all *Sdic* paralogs in such strains is denoted with orange dots. *Sdic* paralogs are shown from left to right as they are arranged along the cluster from centromere to telomere on the *X* chromosome of each strain. Asterisks indicate paralogs with expression levels significantly different from those expected assuming equal partitioning of *Sdic* expression among all the copies (dashed line) according to Monte Carlo simulations and upon correcting by multiple tests [8]; *, P_{adj}<0.05; **, P_{adj}<0.01. Log_{10}(RPKM) expression values were used as input for the simulations. The lower and upper limits of the 95% confidence intervals of the expected expression of any of the paralogs are indicated with blue and grey dots, respectively. The adjusted *p*-value of each paralog in each strain is provided in Supplementary Table 6, and the source data for this plot are provided in Supplementary Data 4.

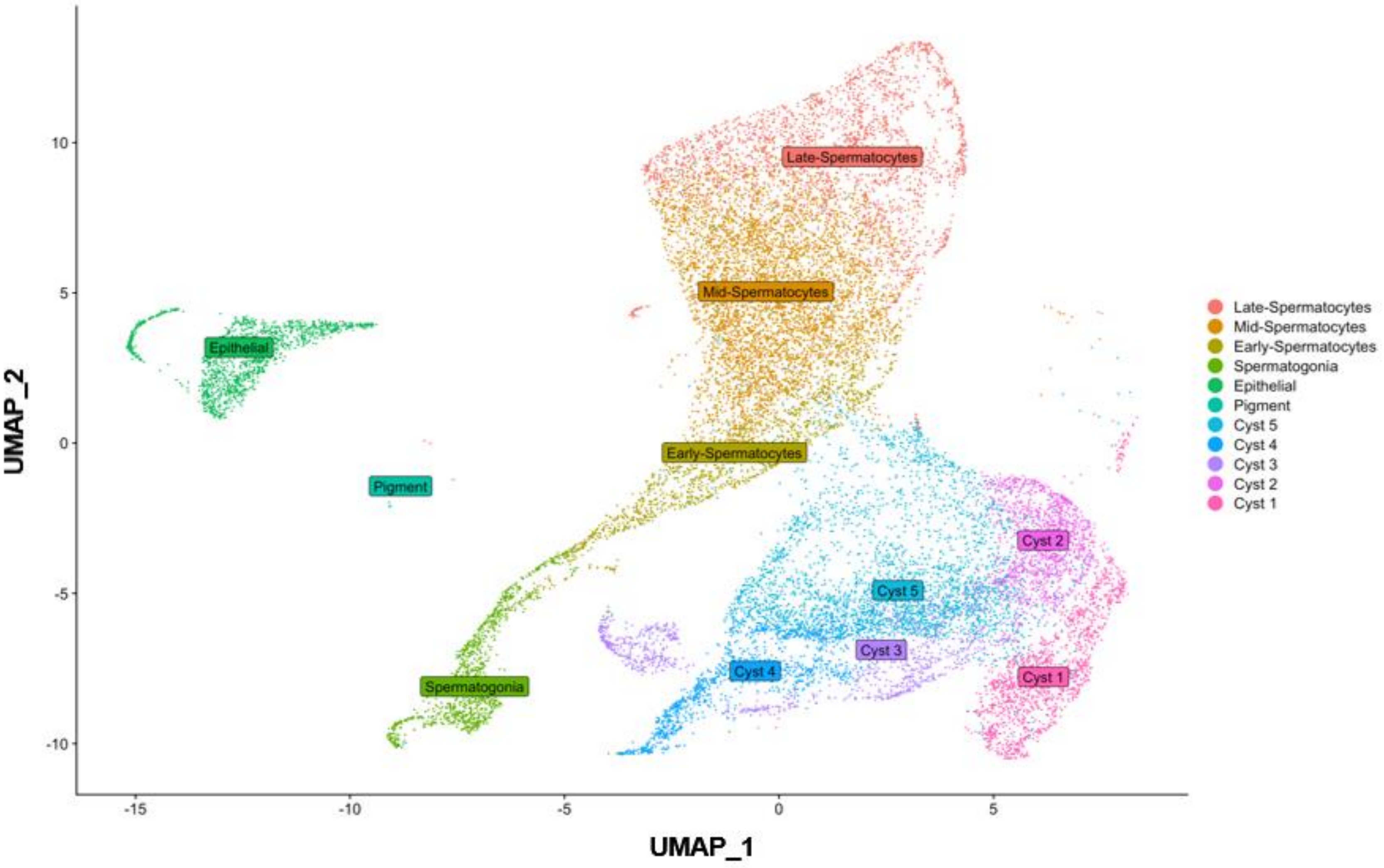

Supplementary Figure 7. UMAP plot showing the patterns of cell clustering in *D. melanogaster* testis of third instar larvae. The different cell types are indicated.

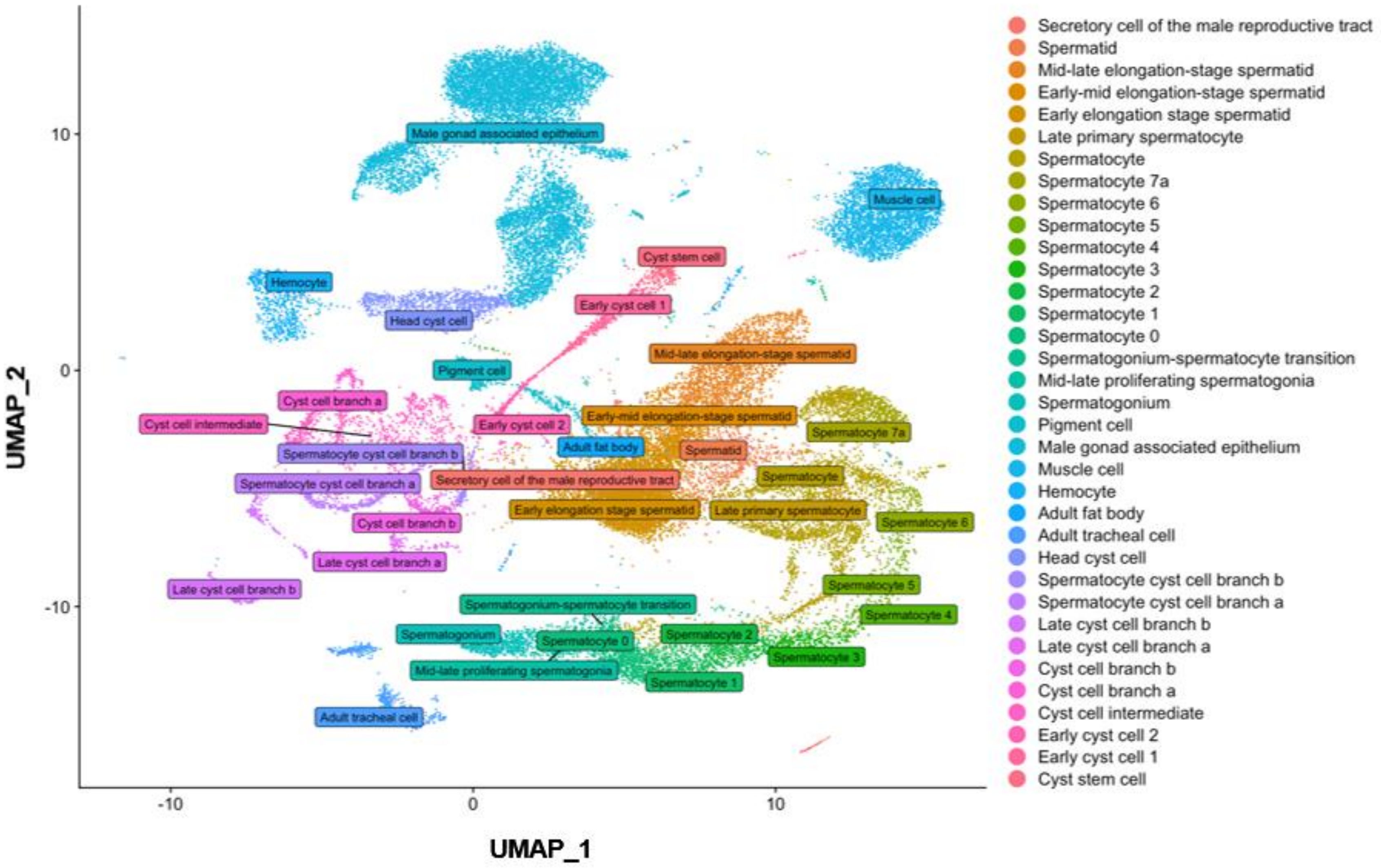

Supplementary Figure 8. UMAP plot showing the patterns of cell clustering in *D. melanogaster* testis of adult individuals. The different cell types are indicated.

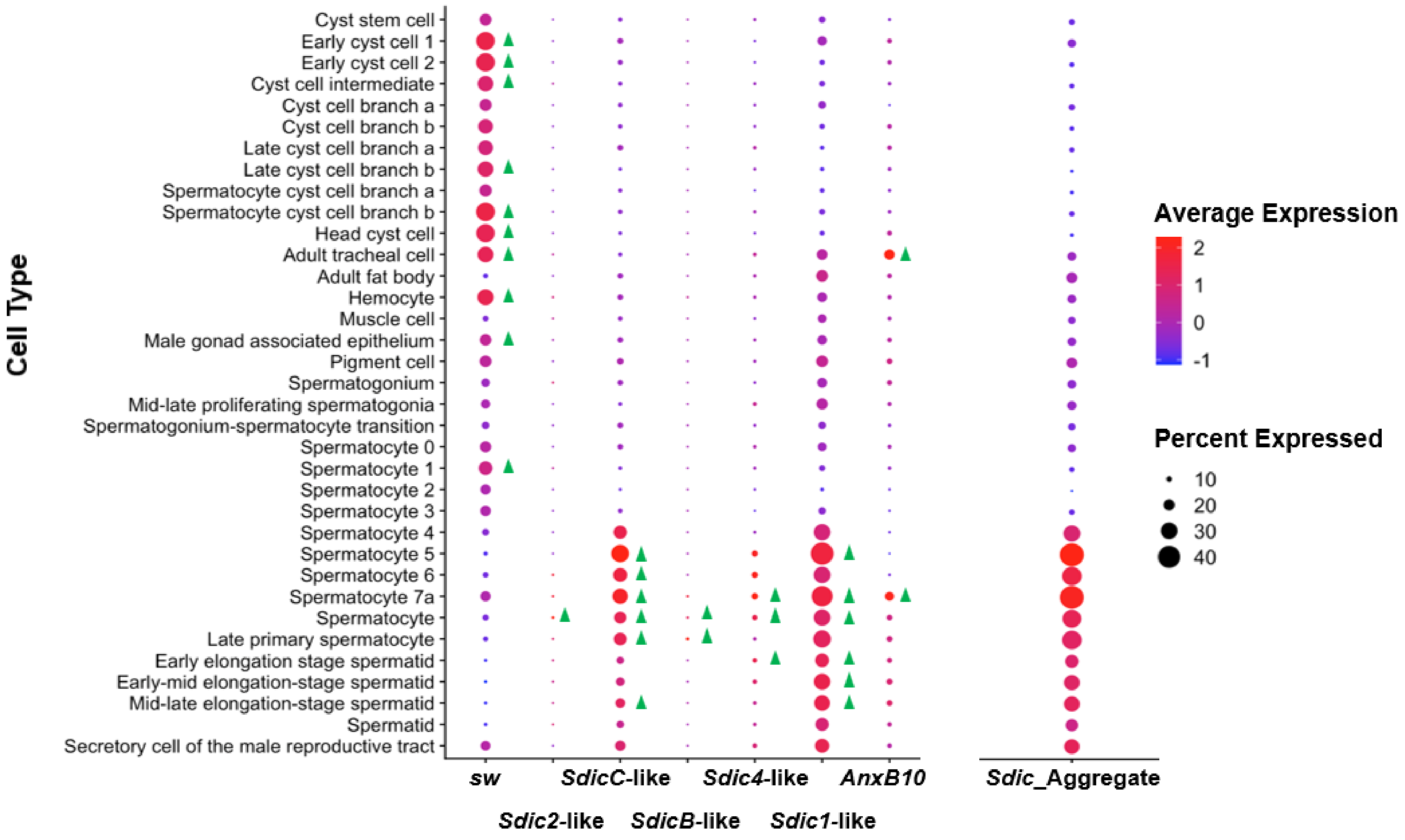

Supplementary Figure 9. Differential expression of *Sdic* paralogs across testis cell populations at two life stages of *D. melanogaster*. Cell types are indicated on the *y*-axis and the different *Sdic* paralogs appear on the *x*-axis. The flanking parental genes *sw* and *AnxB10* are included for the sake of completeness. The order of the genes (from left to right) mirrors that in the genome (from centromere to telomere) of ISO1. This order is not necessarily the same in w^{1118}. The aggregate expression level considering all *Sdic* copies for which we find evidence of expression is shown on the right. Average expression is color-coded, reflecting the z-scores calculated by scaling the log(corrected counts), which are in turn computed using the SCTransform v2 regularization. The diameter of the bubbles denotes the percentage of cells within a given cluster that expresses the respective gene. Green triangles indicate cell types for which a particular paralog showed significantly higher expression relative to the average expression level in the rest of the cell types (P_{adj}<0.05). The paralog *Sdic3*-like is not represented as no detectable expression was found. Normalized expression levels are provided in Supplementary Data 5.

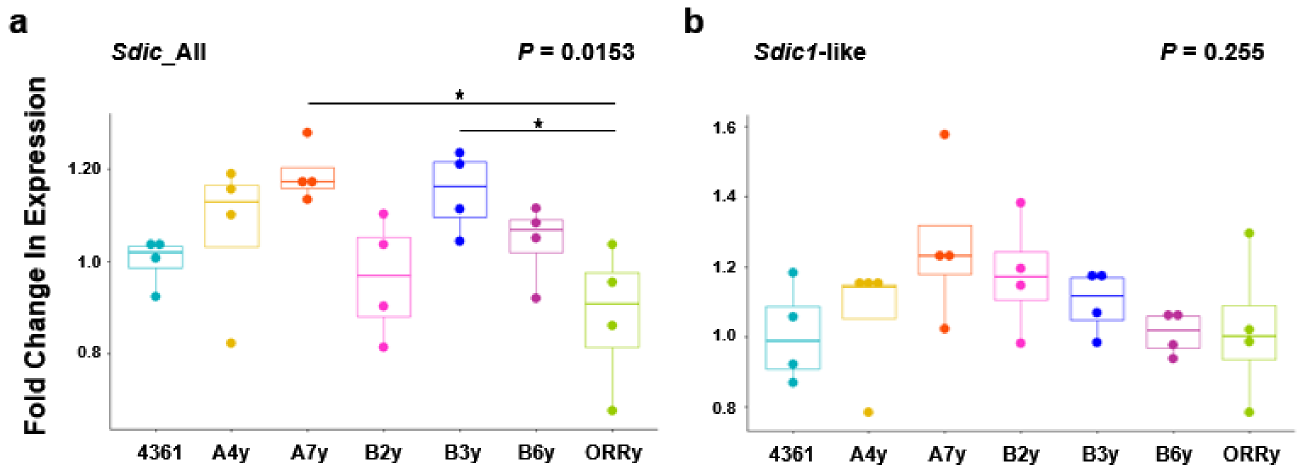

Supplementary Figure 10. *Y* chromosome origin impacts the combined expression of the *Sdic* paralogs, but not *Sdic1*-like alone, across male whole-body samples. Expression levels of all *Sdic* paralogs considered jointly (**a**) and *Sdic1*-like (**b**) in male whole-bodies from each of the *Y* chromosome substitution strains from panel II are plotted as their fold change relative to the strain 4361 (value of 1 on the *y*-axis), which was used as calibrator, in qRT-PCR assays. Boxes represent the interquartile range (IQR) around the median (horizontal black line) and whiskers extend to 1.5 times the IQR. One-way ANOVA *P*-values are boxed on top. Black bars connect significant pairwise comparisons (Tukey HSD); *, $P<0.05$. Statistical values for all comparisons are listed in Supplementary Table 10 and normalized expression values (n=4 biological) per strain are provided in Supplementary Data 6.

Supplementary Table 1. *D. melanogaster* strains used in this study

Strain ID	Strain origin	*Sdic* CN †	Expression Assay ‡
**I. *Sdic* CNV panel**			
ISO1	Reference strain	6	RNA-seq (T)
A4	Kariba Dam, South Africa	5	RNA-seq (T)
A7	Ken-Ting, Taiwan	4	RNA-seq (T)
B6	Ica, Peru	3	RNA-seq (T)
**II. *Y* chromosome substitution panel**			
4361: *y*[1]; *bw*[1]; *e*[4]; *ci*[1] *ey*[R]	Bloomington Stock Center	?	qRT-PCR WB, RNA-seq (T, AG)
A4y	Ranz Lab	?	qRT-PCR WB, RNA-seq (T)
A7y	Ranz Lab	?	qRT-PCR WB, RNA-seq (T, AG)
B2y	Ranz Lab	?	qRT-PCR WB
B3y	Ranz Lab	?	qRT-PCR WB
B6y	Ranz Lab	?	qRT-PCR WB, RNA-seq (T)
ORRy	Ranz Lab	?	qRT-PCR WB

† CN, copy number (Clifton et al. 2020); ?, unknown.
‡ Sample profiled: WB, whole-body; T, testis; AG, male accessory gland.

Supplementary Table 2. Number of sequencing reads obtained in the RNA-seq libraries generated

Strain ID	Replicate ID	Testis	Male Accessory Gland
**I. *Sdic* CNV panel**			
ISO1	1	100,227,657	NA
	2	76,600,012	NA
	3	87,240,106	NA
A4	1	91,152,702	NA
	2	92,604,159	NA
	3	84,371,915	NA
A7	1	97,971,222	NA
	2	82,558,848	NA
	3	86,305,799	NA
B6	1	90,150,311	NA
	2	85,387,534	NA
	3	95,598,040	NA
**II. *Y* chromosome substitution panel**			
4361	1	111,342,175	107,897,454
	2	109,620,102	111,330,037
	3	103,719,660	106,289,848
A4y	1	94,710,777	NA
	2	97,794,319	NA
	3	98,415,754	NA
A7y	1	116,629,936	118,126,878
	2	101,031,864	124,991,816
	3	106,626,300	116,538,336
B6y	1	91,537,903	NA
	2	115,974,917	NA
	3	100,374,763	NA

Supplementary Table 3. Diagnostic motifs used to detect expression in RNA-seq datasets

Gene ID	Core Diagnostic Motif*	Extended Diagnostic Motif*
*sw*_R2	CCGGCGTCGCGAGAAGGAGA	CTGAGCTGGAACGCAAGAAGGCCAAGTTGGCCGCCCTGCGCGAGGAGAAGGATCG**CCGGCGTCGCGAGAAGGAGA**TCAAGGACATGGAGGAGGCGGCCGGTCGCATTGGCGGCGGAGCAGGCATCGACAA
*sw*_R1	TCTCCTTCTCGCGACGCCGG	TTGTCGATGCCTGCTCCGCCGCCAATGCGACCGGCCGCCTCCTCCATGTCCTTGA**TCTCCTTCTCGCGACGCCGG**CGATCCTTCTCCTCGCGCAGGGCGGCCAACTTGGCCTTCTTGCGTTCCAGCTCAG
*Sdic*_All_R2	TCAACGTATTCTACTTTGAG	AGCTTAGTATTACACAGATTGGACAATGGGCTTAGTACTGATTAAGTTTTTACGA**TCAACGTATTCTACTTTGAG**CGGCGGAAAGAAACAGCCTCTCAACCTAAGCGTCTACAATGTGCAGGCTACGAAC
*Sdic*_All_R1	CTCAAAGTAGAATACGTTGA	GTTCGTAGCCTGCACATTGTAGACGCTTAGGTTGAGAGGCTGTTTCTTTCCGCCG**CTCAAAGTAGAATACGTTGA**TCGTAAAAACTTAATCAGTACTAAGCCCATTGTCCAATCTGTGTAATACTAAGCT
Sdic1-like_R2	TTTGAGCAGTACATCGCCTG	CATTGACTGGACCATCAAGCTCTGGTCGCTAAAGGACACCAAGCCGCTGTACTCC**TTTGAGCAGTACATCGCCTG**GTCGCCCGTGCGACGGCAGCGGCCGCCTGGACCTGATAAAACTCAACCCAGACAC
Sdic1-like_R1	CAGGCGATGTACTGCTCAAA	GTGTCTGGGTTGAGTTTTATCAGGTCCAGGCGGCCGCTGCCGTCGCACGGGCGAC**CAGGCGATGTACTGCTCAAA**GGAGTACAGCGGCTTGGTGTCCTTTAGCGACCAGAGCTTGATGGTCCAGTCAATG
ISO1_*Sdic2*_R2	CAATAGCGTGGTGATGGGCA	AGCGCCAGTCTAAGGCCATTGCCATTACATCGATGGCCTTCCCGGCCAACGAGAT**CAATAGCGTGGTGATGGGCA**GTGAGGACGGCTACGTCTACTCCGCCTCGCGCCACGGCCTGCGCTCCGGGGTCAA
ISO1_*Sdic2*_R1	TGCCCATCACCACGCTATTG	TTGACCCCGGAGCGCAGGCCGTGGCGCGAGGCGGAGTAGACGTAGCCGTCCTCAC**TGCCCATCACCACGCTATTG**ATCTCGTTGGCCGGGAAGGCCATCGATGTAATGGCAATGGCCTTAGACTGGCGCT
ISO1_*SdicC*_R2	TCCCCAAGCTGGTGGTGGGC	GACGAGCGCTGGTCGAAGAACCGCTGCATCACCAGCATGGACTGGTCCACCCACT**TCCCCAAGCTGGTGGTGGGC**TCGTACCACAACAACGAGGAGAGTCCGAACGAGCCGGACGGCGTGGTGATGGTGT
ISO1_*SdicC*_R1	GCCCACCACCAGCTTGGGGA	ACACCATCACCACGCCGTCCGGCTCGTTCGGACTCTCCTCGTTGTTGTGGTACGA**GCCCACCACCAGCTTGGGGA**AGTGGGTGGACCAGTCCATGCTGGTGATGCAGCGGTTCTTCGACCAGCGCTCGTC
ISO1_*SdicB*_R2	TGCTACATATTATATTCAAC	TTTATTGTTTATTCGACTGTCGGGCAGGCTGAAAGCAACACATAAATAAATTAAA**TGCTACATATTATATTCAAC**AACGTATTAAGCGTGGAAGTGGGGGCTTGGGAATGAAGGTACGAGGACTGAAGAT
ISO1_*SdicB*_R1	GTTGAATATAATATGTAGCA	ATCTTCAGTCCTCGTACCTTCATTCCCAAGCCCCCACTTCCACGCTTAATACGTT**GTTGAATATAATATGTAGCA**TTTAATTTATTTATGTGTTGCTTTCAGCCTGCCCGACAGTCGAATAAACAATAAA
ISO1_*Sdic3*_R2	TAGCCCAGAACTCAAAACTC	CTTGAATGAAATTTAATTTGTATTTTTGTATCTTTTGTGATCCCGCTACTGTGTA**TAGCCCAGAACTCAAAACTC**AACCGCAGTCCAAGTGCTCAGAATCACTCCAAAGCCCCCAAGAAATCAGGGGAAA
ISO1_*Sdic3*_R1	GAGTTTTGAGTTCTGGGCTA	TTTCCCCTGATTTCTTGGGGGCTTTGGAGTGATTCTGAGCACTTGGACTGCGGTT**GAGTTTTGAGTTCTGGGCTA**TACACAGTAGCGGGATCACAAAAGATACAAAAATACAAATTAAATTTCATTCAAG
ISO1_*Sdic4*_R2	AACACCCATCTTAGTGAGAT	GTACGACGTGGCCGAGAACCTGGCGCAGCCATCGCGCGACGAATGGTCGCGGTTC**AACACCCATCTTAGTGAGAT**CAAGATGAACCAGAGCGATGAGGTCTAGGACGATATAGTTAACTGGTAGTTGAGA
ISO1_*Sdic4*_R1	ATCTCACTAAGATGGGTGTT	TCTCAACTACCAGTTAACTATATCGTCCTAGACCTCATCGCTCTGGTTCATCTTG**ATCTCACTAAGATGGGTGTT**GAACCGCGACCATTCGTCGCGCGATGGCTGCGCCAGGTTCTCGGCCACGTCGTAC
A4_*SdicI*_R2	GCGCCAGTCTAAGGCCATTG	GCTCCTGGTCGCTGGACATGCTGTCGCAACCACAGGACACGCTGGAGCTGCAGCA**GCGCCAGTCTAAGGCCATTG**CCATTACATCGATGGCCTTCCCGGCCAACGAGATCAATAGCCTGGTGATGGGCAG
A4_*SdicI*_R1	CAATGGCCTTAGACTGGCGC	CTGCCCATCACCAGGCTATTGATCTCGTTGGCCGGGAAGGCCATCGATGTAATGG**CAATGGCCTTAGACTGGCGC**TGCTGCAGCTCCAGCGTGTCCTGTGGTTGCGACAGCATGTCCAGCGACCAGGAGC
A4_*SdicII*_R2	TTTCCCAGTTCTGTTCCCAC	TGTAACTGTAACTGTATTATTTTGTTACTCAATATTGGTTTCATTTCATAGCTAT**TTTCCCAGTTCTGTTCCCAC**CAAAAATCGCAACCAAATTGGCTATTCCGACTCCCCGGAAATTTGATTGGTGGTG
A4_*SdicII*_R1	GTGGGAACAGAACTGGGAAA	CACCACCAATCAAATTTCCGGGGAGTCGGAATAGCCAATTTGGTTGCGATTTTTG**GTGGGAACAGAACTGGGAAA**ATAGCTATGAAATGAAACCAATATTGAGTAACAAAATAATACAGTTACAGTTACA
A4_*SdicIII*_R2	CATCGCGCGACGAGATCAAG	GGCGACGAGGCCGGCAAGCTGTACGTGTACGACGTGGCCGAGAACCTGGCGCAGC**CATCGCGCGACGAGATCAAG**ATGAACCAGAGCGATGAGGTCTAGGACGATATAGTTAACTGGTAGTTGAGAGTCG
A4_*SdicIII*_R1	CTTGATCTCGTCGCGCGATG	CGACTCTCAACTACCAGTTAACTATATCGTCCTAGACCTCATCGCTCTGGTTCAT**CTTGATCTCGTCGCGCGATG**GCTGCGCCAGGTTCTCGGCCACGTCGTACACGTACAGCTTGCCGGCCTCGTCGCC
A4_*SdicIV*_R2	AGGCCGTCAAGCTGTACGTG	GCCCTTAACCGCGTCTCTTGGACCCCATCCGGTCTGCACGTGTGCATCGGCGACG**AGGCCGTCAAGCTGTACGTG**TACGACGTGGCCGAGAACCTGGCGCAGCCATCGCGCGACGAATGGTCGCGGTTCA
A4_*SdicIV*_R1	CACGTACAGCTTGACGGCCT	TGAACCGCGACCATTCGTCGCGCGATGGCTGCGCCAGGTTCTCGGCCACGTCGTA**CACGTACAGCTTGACGGCCT**CGTCGCCGATGCACACGTGCAGACCGGATGGGGTCCAAGAGACGCGGTTAAGGGC
A7_*SdicI*_R2	AATAGCGTGGTGATGGGCAG	GCGCCAGTCTAAGGCCATTGCCATTACATCGATGGCCTTCCCGGCCAACGAGATC**AATAGCGTGGTGATGGGCAG**TGAGGACGGCTACGTCTACTCCGCCTCGCGCCACGGCCTGCGCTCCGGGGTCAAC
A7_*SdicI*_R1	CTGCCCATCACCACGCTATT	GTTGACCCCGGAGCGCAGGCCGTGGCGCGAGGCGGAGTAGACGTAGCCGTCCTCA**CTGCCCATCACCACGCTATT**GATCTCGTTGGCCGGGAAGGCCATCGATGTAATGGCAATGGCCTTAGACTGGCGC
A7_*SdicII*_R2	AGACACGAAGGTGCCGACCG	CACTCTTCGCCGCCGTCGACGGCAGCGGCCGCCTGGACCTGTGGAACCTCAACCA**AGACACGAAGGTGCCGACCG**CCTCGATTGTCGTGGCGGGAGCACCAGCCCTTAACCGCGTCTCTTGGACCCCATC
A7_*SdicII*_R1	CGGTCGGCACCTTCGTGTCT	GATGGGGTCCAAGAGACGCGGTTAAGGGCTGGTGCTCCCGCCACGACAATCGAGG**CGGTCGGCACCTTCGTGTCT**TGGTTGAGGTTCCACAGGTCCAGGCGGCCGCTGCCGTCGACGGCGGCGAAGAGTG
A7_*SdicIII*_R2	AGGACGTCCCCCCGGCCATC	GGATTCACCTCCAAGCTGCCACCGGGCTATCTCACCCACGGCCTGCCCACCGTTA**AGGACGTCCCCCCGGCCATC**ACACCACTCGAGATCAAGAAGGAGACTGAAGTGAAGAAGGAGGTCAACGAGCTGT
A7_*SdicIII*_R1	GATGGCCGGGGGGACGTCCT	ACAGCTCGTTGACCTCCTTCTTCACTTCAGTCTCCTTCTTGATCTCGAGTGGTGT**GATGGCCGGGGGGACGTCCT**TAACGGTGGGCAGGCCGTGGGTGAGATAGCCCGGTGGCAGCTTGGAGGTGAATCC
B6_*SdicI*_R2	CATCGCGCGACGAGATCAAG	GGCGACGAGGCCGGCAAGCTGTACGTGTACGACGTGGCCGAGAACCTGGCGCAGC**CATCGCGCGACGAGATCAAG**ATGAACCAGAGCGATGAGGTCTAGGACGATATAGTTAACTGGTAGTTGAGAGTCG
B6_*SdicI*_R1	CTTGATCTCGTCGCGCGATG	CGACTCTCAACTACCAGTTAACTATATCGTCCTAGACCTCATCGCTCTGGTTCAT**CTTGATCTCGTCGCGCGATG**GCTGCGCCAGGTTCTCGGCCACGTCGTACACGTACAGCTTGCCGGCCTCGTCGCC
B6_*SdicII*_R2	CATCTTAGTGAGATCAAGAT	CGTGGCCGAGAACCTGGCGCAGCCATCGCGCGACGAATGGTCGCGGTTCAACACC**CATCTTAGTGAGATCAAGAT**GAACCAGAGCGATGAGGTCTAGGACGATATAGTTAACTGGTAGTTGAGAGTCGGT
B6_*SdicII*_R1	ATCTTGATCTCACTAAGATG	ACCGACTCTCAACTACCAGTTAACTATATCGTCCTAGACCTCATCGCTCTGGTTC**ATCTTGATCTCACTAAGATG**GGTGTTGAACCGCGACCATTCGTCGCGCGATGGCTGCGCCAGGTTCTCGGCCACG

*Sequences shown correspond with the sense strand. Both the sense and its reverse complement sequences were used. In the case of different *Sdic* paralogs, the name of their resident strain appears first.

Supplementary Table 4. Test for the differences in total *Sdic* expression in testis across strains in panel I

Test	Contrast	*P*
One-way ANOVA	Total *Sdic* across strains	7.520E-04
Tukey HSD	A4-ISO1	1.078E-01
Tukey HSD	A7-ISO1	9.986E-03
Tukey HSD	B6-ISO1	5.264E-04
Tukey HSD	A7-A4	3.696E-01
Tukey HSD	B6-A4	1.093E-02
Tukey HSD	B6-A7	1.189E-01

Supplementary Table 5. Test for the differences in testis expression levels among paralogs in four strains of *D. melanogaster*

Test	Contrast	*P*
One-way ANOVA	ISO1 paralogs	8.080E-13
Tukey HSD	*SdicC-Sdic2*	1.072E-02
Tukey HSD	*SdicB-Sdic2*	<2E-16
Tukey HSD	*Sdic3-Sdic2*	1.304E-02
Tukey HSD	*Sdic4-Sdic2*	6.549E-02
Tukey HSD	*Sdic1-Sdic2*	1.593E-03
Tukey HSD	*SdicB-SdicC*	<2E-16
Tukey HSD	*Sdic3-SdicC*	1
Tukey HSD	*Sdic4-SdicC*	8.819E-01
Tukey HSD	*Sdic1-SdicC*	8.411E-01
Tukey HSD	*Sdic3-SdicB*	<2E-16
Tukey HSD	*Sdic4-SdicB*	<2E-16
Tukey HSD	*Sdic1-SdicB*	<2E-16
Tukey HSD	*Sdic4-Sdic3*	9.224E-01
Tukey HSD	*Sdic1-Sdic3*	7.850E-01
Tukey HSD	*Sdic1-Sdic4*	2.851E-01
One-way ANOVA	A4 paralogs	1.110E-06
Tukey HSD	*SdicII-SdicI*	7.199E-02
Tukey HSD	*SdicIII-SdicI*	7.100E-06
Tukey HSD	*SdicIV-SdicI*	7.800E-06
Tukey HSD	*SdicV-SdicI*	5.500E-06
Tukey HSD	*SdicIII-SdicII*	1.455E-04
Tukey HSD	*SdicIV-SdicII*	1.652E-04
Tukey HSD	*SdicV-SdicII*	1.034E-04
Tukey HSD	*SdicIV-SdicIII*	1
Tukey HSD	*SdicV -SdicIII*	9.977E-01
Tukey HSD	*SdicV-SdicIV*	9.925E-01
One-way ANOVA	A7 paralogs	9.470E-04
Tukey HSD	*SdicII-SdicI*	1.042E-01
Tukey HSD	*SdicIII-SdicI*	9.044E-04
Tukey HSD	*SdicIV-SdicI*	4.097E-03
Tukey HSD	*SdicIII-SdicII*	2.299E-02
Tukey HSD	*SdicIV-SdicII*	1.506E-01
Tukey HSD	*SdicIV -SdicIII*	5.635E-01
One-way ANOVA	B6 paralogs	8.090E-02

Supplementary Table 6. Test for an even contribution of *Sdic* paralogs to total *Sdic* expression in testis across four strains of *D. melanogaster*

Strain_Paralog	Over Contribution (P_{adj})	Under Contribution (P_{adj})
ISO1_*Sdic2*	0.9672	0.8732
ISO1_*SdicC*	0.4683	1
ISO1_*SdicB*	1	0.0048
ISO1_*Sdic3*	0.4683	1
ISO1_*Sdic4*	0.811	0.9946
ISO1_*Sdic1*	0.0048	1
A4_*SdicI*	1	0.019
A4_*SdicII*	1	0.2197
A4_*SdicIII*	0.233	1
A4_*SdicIV*	0.233	1
A4_*SdicV*	0.2197	1
A7_*SdicI*	1	0.0408
A7_*SdicII*	1	0.535
A7_*SdicIII*	0.0776	1
A7_*SdicIV*	0.3515	1
B6_*SdicI*	0.775	0.9358
B6_*SdicII*	1	0.0588
B6_*SdicIII*	0.2172	1

According to Monte Carlo simulations (n=10,000) per strain, followed by multiple test correction [8]. Significant cases of higher or lower expression relative to that expected under an equal portioning of the total *Sdic* expression level (P_{adj}<0.05) are indicated in blue and red, respectively.

Supplementary Table 7. Marker genes used to associate particular clusters in UMAP plots with particular testis cell types in third instar larvae (L3)

Cluster #	Gene *	Cell Type (Symbol)
0, 4, 6, 8, 9	*Nrt, wnt4, bnb, fax, Sap-r, rdo, Tep2, Nrg, kek1*	Cyst (C1, C2, C3, C4, C5)
12, 17	*Tok*	Pigment (P)
7, 14	*nord, Piezo, Fas3*	Epithelial (T)
10	*aub, vas, p53, Dek*	Spermatogonia (G)
5, 16	*sa, tbrd-1*	Early-stage spermatocyte (E)
1, 2	*dj, ocn, dpr17, CycB, Mst87F, twin, fest, CG3927*	Mid-stage spermatocyte (M)
3, 13	*dj, ocn, bol, spir, fzo, Mst87F, twin*	Late-stage spermatocyte (L)
11, 12, 15	*NA*	Unknown (UK)

* As previously reported [9].

Supplementary Table 8. Salient features of the sc- and snRNA-seq datasets analyzed

Metric	L3 Testis †	Adult Testis †
Number of Estimated Cells *	21,000	44,621
Median Genes/Cell	763	1,444
Total Reads Aligned	95,630,491	215,233,728
Average Reads/Cell	4,554	4,824

* Post-alignment, filtering, and doublet removal.

† Upon combining all biological replicates.

Supplementary Table 9. Pearson's correlations among the expression levels of *Sdic* paralogs and its flanking parental genes *sw* and *AnxB10* across cell types at two different developmental stages

Stage		Genes						
		AnxB10	***Sdic1*-like**	***Sdic4*-like**	***SdicB*-like**	***SdicC*-like**	***Sdic2*-like**	***sw***
L3	***AnxB10***	-	0.0113	-0.0220		-0.0083		0.7807 *
	***Sdic1*-like**		-	0.9399 ****		0.9870 ****		-0.1967
	***Sdic4*-like**			-		0.9585 ****		-0.2184
	***SdicB*-like**							-0.1754
	***SdicC*-like**					-		-0.4632
	***Sdic2*-like**							
	sw							-
Adult	***AnxB10***	-	0.3717 *	0.3251	0.2824	0.1373	0.3960 *	0.0273
	***Sdic1*-like**		-	0.7872 ****	0.3642 *	0.8754 ****	0.5194 **	-0.7144 ****
	***Sdic4*-like**			-	0.2228	0.7966 ****	0.6059 ***	-0.4896 **
	***SdicB*-like**				-	0.4193 *	0.4723 **	-0.2166
	***SdicC*-like**					-	0.5291 **	-0.6190 ****
	***Sdic2*-like**						-	-0.3847 *
	sw							-

Only paralogs for which there is certainty of their existence in w^{1118} are included.

Significant Pearson's correlations are color-coded (positive, blue; negative, red). Correlation probabilities were corrected for multiple tests [8]. * P_{adj}<0.05; ** P_{adj}<0.01; *** P_{adj}<0.001; **** P_{adj}<0.0001.

Supplementary Table 10. Test for the differences in total *Sdic* and *Sdic1*-like expression in male whole-body samples across panel II of strains using qRT-PCR

Test	Contrast	*P*
One-way ANOVA	Total *Sdic* expression across strains	1.53E-02
Tukey HSD	A4y-4361	9.79E-01
Tukey HSD	A7y-4361	2.71E-01
Tukey HSD	B2y-4361	9.99E-01
Tukey HSD	B3y-4361	5.29E-01
Tukey HSD	B6y-4361	9.98E-01
Tukey HSD	ORRy-4361	7.55E-01
Tukey HSD	A7y-A4y	7.34E-01
Tukey HSD	B2y-A4y	8.50E-01
Tukey HSD	B3y-A4y	9.42E-01
Tukey HSD	B6y-A4y	1
Tukey HSD	ORRy-A4y	2.88E-01
Tukey HSD	B2y-A7y	1.22E-01
Tukey HSD	B3y-A7y	9.99E-01
Tukey HSD	B6y-A7y	5.43E-01
Tukey HSD	ORRy-A7y	1.48E-02
Tukey HSD	B3y-B2y	2.83E-01
Tukey HSD	B6y-B2y	9.55E-01
Tukey HSD	ORRy-B2y	9.45E-01
Tukey HSD	B6y-B3y	8.24E-01
Tukey HSD	ORRy-B3y	4.24E-02
Tukey HSD	ORRy-B6y	4.51E-01
One-way ANOVA	*Sdic1*-like expression across strains	2.55E-01

Supplementary Table 11. Test for the differences in total *Sdic* expression in testis and accessory gland across panel II of strains using RNA-seq

Test	Contrast	*P*
One-way ANOVA	Total *Sdic* expression in testis across strains	1.820E-03
Tukey HSD	A4y-4361	9.862E-01
Tukey HSD	A7y-4361	2.251E-03
Tukey HSD	B6y-4361	2.163E-01
Tukey HSD	A7y-A4y	3.319E-03
Tukey HSD	B6y-A4y	3.322E-01
Tukey HSD	B6y-A7y	3.496E-02
One-way ANOVA	Total *Sdic* expression in accessory gland across strains	4.020E-02

Supplementary Table 12. PCR primer sets and conditions used in amplification experiments

Amplicon	Ta (C)	Primer efficiency, R^2	Size (nt)	Forward Primer (5'-3')	Reverse Primer (5'-3')	Experiment
*Sdic*_All	60	96.8%, 0.995	76	CGTATTCTACTTTGAGCGGCG‡	GGAATGTTCGTAGCCTGCAC	qRT-PCR
Sdic1-like	60	92.7%, 0.999	195	TCTGGTCGCTAAAGGACACC‡	CGTCGTACACGTACAGCTTGC	qRT-PCR
clot	60	99.9%, 0.988	82	GAGCGGGCATACTGGAAG	GCAACAGAGTGGGCAAGAAG	qRT-PCR
Gapdh2 †	52	n/a	761	CAAGCAAGCCGATAGATAAAC‡	GTCAAATCGACCACGGAAA	RT-PCR

Ta, annealing temperature.
† Used as a control of successful reverse transcriptase reactions.
‡ Priming site spans a splice junction.

SUPPLEMENTARY REFERENCES

1. Leader, D.P., Krause, S.A., Pandit, A., Davies, S.A. & Dow, J.A.T. FlyAtlas 2: a new version of the Drosophila melanogaster expression atlas with RNA-Seq, miRNA-Seq and sex-specific data. *Nucleic Acids Res* **46**, D809-D815 (2018).
2. Brown, J.B. *et al.* Diversity and dynamics of the Drosophila transcriptome. *Nature* **512**, 393-9 (2014).
3. Chen, M.J. *et al.* Integrating RNA-seq and ChIP-seq data to characterize long non-coding RNAs in Drosophila melanogaster. *BMC Genomics* **17**, 220 (2016).
4. Clifton, B.D. *et al.* Rapid Functional and Sequence Differentiation of a Tandemly Repeated Species-Specific Multigene Family in Drosophila. *Mol Biol Evol* **34**, 51-65 (2017).
5. Kumar, S., Stecher, G., Li, M., Knyaz, C. & Tamura, K. MEGA X: Molecular Evolutionary Genetics Analysis across Computing Platforms. *Molecular biology and evolution* **35**, 1547-1549 (2018).
6. Paysan-Lafosse, T. *et al.* InterPro in 2022. *Nucleic Acids Res* **51**, D418-D427 (2023).
7. Clifton, B.D. *et al.* Understanding the Early Evolutionary Stages of a Tandem Drosophilamelanogaster-Specific Gene Family: A Structural and Functional Population Study. *Mol Biol Evol* **37**, 2584-2600 (2020).
8. Benjamini, Y. & Hochberg, Y. Controlling the False Discovery Rate - a Practical and Powerful Approach to Multiple Testing. *Journal of the Royal Statistical Society Series B-Methodological* **57**, 289-300 (1995).
9. Mahadevaraju, S. *et al.* Dynamic sex chromosome expression in Drosophila male germ cells. *Nat Commun* **12**, 892 (2021).
