# Supplementary material for: Paralog transcriptional differentiation in the D. melanogaster-specific gene family Sdic across populations and spermatogenesis stages
Source: Commun Biol. 2023 Oct 20;6:1069. doi: 10.1038/s42003-023-05427-4 (PMC10589255; doi:10.1038/s42003-023-05427-4)
Supplement: Supplementary file 2 — Description of Additional Supplementary Files [file 42003_2023_5427_MOESM2_ESM.docx]

**Description of Additional Supplementary Files**

**File name:** Supplementary Data 1

**Description:** Total *Sdic* expression in RNA-seq experiments across different developmental stages and anatomical parts of *D. melanogaster* (source data Supplementary Fig. 2).

**File name:** Supplementary Data 2

**Description:** Total testis *Sdic* expression in RNA-seq experiments across four *D. melanogaster* strains with variable number of *Sdic* copies (source data Fig. 2).

**File name:** Supplementary Data 3

**Description:** Testis expression of *Sdic* paralogs in RNA-seq experiments across four *D. melanogaster* strains (source data Fig. 3).

**File name:** Supplementary Data 4

**Description:** Test for a non-equal contribution to the total Sdic expression in RNA-seq experiments across four D. melanogaster strains.

**File name:** Supplementary Data 5

**Description:** Normalized expression level in testis of different *Sdic* paralogs and their parental flanking genes *sw* and *AnxB10* (source data Fig. 4 and Supplementary Fig. 9).

**File name:** Supplementary Data 6

**Description:** Ct values in two qRT-PCR expression surveys performed in whole-body males from seven *Y*-chromosome substitution lines of *D. melanogaster* (source data Supplementary Fig. 10).

**File name:** Supplementary Data 7

**Description:** Total testis expression of *Sdic* paralogs in RNA-seq experiments across four *Y*-chromosome substitution lines of *D. melanogaster* (source data Fig. 5).
