# Supplementary material for: Paralog transcriptional differentiation in the D. melanogaster-specific gene family Sdic across populations and spermatogenesis stages
Source: Commun Biol. 2023 Oct 20;6:1069. doi: 10.1038/s42003-023-05427-4 (PMC10589255; doi:10.1038/s42003-023-05427-4)
Supplement: Supplementary file 4 — Reporting Summary [file 42003_2023_5427_MOESM4_ESM.pdf]

Corresponding author(s): Jose Ranz & Bryan Clifton

Last updated by author(s): Oct 4, 2023

Reporting Summary

Nature Portfolio wishes to improve the reproducibility of the work that we publish. This form provides structure for consistency and transparency in reporting. For further information on Nature Portfolio policies, see our Editorial Policies and the Editorial Policy Checklist.

Statistics

For all statistical analyses, confirm that the following items are present in the figure legend, table legend, main text, or Methods section.

n/a	Confirmed	
☐	☒	The exact sample size (*n*) for each experimental group/condition, given as a discrete number and unit of measurement
☐	☒	A statement on whether measurements were taken from distinct samples or whether the same sample was measured repeatedly
☐	☒	The statistical test(s) used AND whether they are one- or two-sided<br>*Only common tests should be described solely by name; describe more complex techniques in the Methods section.*
☒	☐	A description of all covariates tested
☐	☒	A description of any assumptions or corrections, such as tests of normality and adjustment for multiple comparisons
☐	☒	A full description of the statistical parameters including central tendency (e.g. means) or other basic estimates (e.g. regression coefficient) AND variation (e.g. standard deviation) or associated estimates of uncertainty (e.g. confidence intervals)
☐	☒	For null hypothesis testing, the test statistic (e.g. *F*, *t*, *r*) with confidence intervals, effect sizes, degrees of freedom and *P* value noted<br>*Give P values as exact values whenever suitable.*
☒	☐	For Bayesian analysis, information on the choice of priors and Markov chain Monte Carlo settings
☒	☐	For hierarchical and complex designs, identification of the appropriate level for tests and full reporting of outcomes
☒	☐	Estimates of effect sizes (e.g. Cohen's *d*, Pearson's *r*), indicating how they were calculated

Our web collection on statistics for biologists contains articles on many of the points above.

Software and code

Policy information about availability of computer code

Data collection	Most data analyzed are original to this work. The Methods section describes how the RNA-seq and qRT-PCR were obtained. In addition, we used and indicate several other external data sets to this manuscript. These data sets correspond to single-cell RNAseq experiments.
Data analysis	No novel algorithm was designed for this project. We described all the tools used in the Methods section. HTStream was used to process RNA-seq reads derived from bulk-tissue experiments. A custom Bash script used in previous publications was implemented to quantify RNA-seq reads related to Sdic paralogs or all the multigene family. This script is available in Zenodo. For the statistical analysis of these data, we used publicly available R/Bioconductor packages. The R packages were called from within R scripts that we wrote. Although they are not part of any formal pipeline, any of the used in-home scripts can be shared upon request. Data plotting was done in R. Lastly, in the case of the scRNA-seq data analysis, we used a conventional R-based computational pipeline that we described in the Methods. This pipeline allowed us to generate as well the UMAP plots.

For manuscripts utilizing custom algorithms or software that are central to the research but not yet described in published literature, software must be made available to editors and reviewers. We strongly encourage code deposition in a community repository (e.g. GitHub). See the Nature Portfolio guidelines for submitting code & software for further information.

Data

Policy information about availability of data

All manuscripts must include a data availability statement. This statement should provide the following information, where applicable:

- Accession codes, unique identifiers, or web links for publicly available datasets
- A description of any restrictions on data availability
- For clinical datasets or third party data, please ensure that the statement adheres to our policy

All raw sequencing data were deposited as part of the NCBI BioProject PRJNA971348. This project will become publicly available upon acceptance.

Research involving human participants, their data, or biological material

Policy information about studies with human participants or human data. See also policy information about sex, gender (identity/presentation), and sexual orientation and race, ethnicity and racism.

Reporting on sex and gender	*Use the terms sex (biological attribute) and gender (shaped by social and cultural circumstances) carefully in order to avoid confusing both terms. Indicate if findings apply to only one sex or gender; describe whether sex and gender were considered in study design; whether sex and/or gender was determined based on self-reporting or assigned and methods used. Provide in the source data disaggregated sex and gender data, where this information has been collected, and if consent has been obtained for sharing of individual-level data; provide overall numbers in this Reporting Summary. Please state if this information has not been collected. Report sex- and gender-based analyses where performed, justify reasons for lack of sex- and gender-based analysis.*
Reporting on race, ethnicity, or other socially relevant groupings	*Please specify the socially constructed or socially relevant categorization variable(s) used in your manuscript and explain why they were used. Please note that such variables should not be used as proxies for other socially constructed/relevant variables (for example, race or ethnicity should not be used as a proxy for socioeconomic status). Provide clear definitions of the relevant terms used, how they were provided (by the participants/respondents, the researchers, or third parties), and the method(s) used to classify people into the different categories (e.g. self-report, census or administrative data, social media data, etc.) Please provide details about how you controlled for confounding variables in your analyses.*
Population characteristics	*Describe the covariate-relevant population characteristics of the human research participants (e.g. age, genotypic information, past and current diagnosis and treatment categories). If you filled out the behavioural & social sciences study design questions and have nothing to add here, write "See above."*
Recruitment	*Describe how participants were recruited. Outline any potential self-selection bias or other biases that may be present and how these are likely to impact results.*
Ethics oversight	*Identify the organization(s) that approved the study protocol.*

Note that full information on the approval of the study protocol must also be provided in the manuscript.

Field-specific reporting

Please select the one below that is the best fit for your research. If you are not sure, read the appropriate sections before making your selection.

☒ Life sciences ☐ Behavioural & social sciences ☐ Ecological, evolutionary & environmental sciences

For a reference copy of the document with all sections, see nature.com/documents/nr-reporting-summary-flat.pdf

Life sciences study design

All studies must disclose on these points even when the disclosure is negative.

Sample size	Sample sizes implemented were chosen in order to obtain enough RNA material from enough representative number of individuals of each genotype as to avoid any experimental bias. Specifically: 25 male whole-bodies; 100 pairs of accessory glands; and 60 and 100 pairs of testes for the panels of strains I and II, respectively.
Data exclusions	No data was excluded from the analyses.
Replication	Three biological replicates per genotype and type of sample were used in RNA-seq experiments. For qRT-PCR, we used four biological replicates.
Randomization	Random individual flies were collected and distributed across biolocial replicates of any given genotype.
Blinding	Blinding is not relevant to our study.

Reporting for specific materials, systems and methods

We require information from authors about some types of materials, experimental systems and methods used in many studies. Here, indicate whether each material, system or method listed is relevant to your study. If you are not sure if a list item applies to your research, read the appropriate section before selecting a response.

Materials & experimental systems

n/a	Involved in the study
☒	☐ Antibodies
☒	☐ Eukaryotic cell lines
☒	☐ Palaeontology and archaeology
☐	☒ Animals and other organisms
☒	☐ Clinical data
☒	☐ Dual use research of concern
☒	☐ Plants

Methods

n/a	Involved in the study
☒	☐ ChIP-seq
☒	☐ Flow cytometry
☒	☐ MRI-based neuroimaging

Animals and other research organisms

Policy information about studies involving animals; ARRIVE guidelines recommended for reporting animal research, and Sex and Gender in Research

Laboratory animals	All strains correspond to the species Drosophila melanogaster. Tissues from strains ISO1, A4, A7, B6 were dissected from 5d individuals. Whole bodies from strains 4361, A4y, A7y, B2y, B3y, B6y, and ORRy were aged to 5d adult. Tissues from strains 4361, A4y, A7y, and B6y were dissected from 4-6d adult individuals.
Wild animals	NA
Reporting on sex	All data came from males.
Field-collected samples	NA
Ethics oversight	No ethical approval was required for this species.

Note that full information on the approval of the study protocol must also be provided in the manuscript.
